# Supplementary material for: Comparing human and model-based forecasts of COVID-19 in Germany and Poland
Source: PLoS Comput Biol. 2022 Sep 19;18(9):e1010405. doi: 10.1371/journal.pcbi.1010405 (PMC9534421; doi:10.1371/journal.pcbi.1010405)
Supplement: S1 Text — (PDF) [file pcbi.1010405.s001.pdf]

Text S1. Further details on the semi-mechanistic forecasting models

Contents

0.1 Text S1. Further details on the semi-mechanistic forecasting models 1

0.1 Text S1. Further details on the semi-mechanistic forecasting models

0.1.1 Renewal equation model

The model was initialised prior to the first observed data point by assuming constant exponential growth for the mean of assumed delays from infection to case report.

$$I_t = I_0 \exp(rt) \tag{1}$$
$$I_0 \sim \mathcal{LN}(\log I_{obs}, 0.2) \tag{2}$$
$$r \sim \mathcal{LN}(r_{obs}, 0.2) \tag{3}$$

Where I_{obs} and r_{obs} are estimated from the first week of observed data. For the time window of the observed data infections were then modelled by weighting previous infections by the generation time and scaling by the instantaneous reproduction number. These infections were then convolved to cases by date (O_t) and cases by date of report (D_t) using log-normal delay distributions. This model can be defined mathematically as follows,

$$\log R_t = \log R_{t-1} + \text{GP}_t \tag{4}$$
$$I_t = R_t \sum_{\tau=1}^{15} w(\tau|\mu_w, \sigma_w) I_{t-\tau} \tag{5}$$
$$O_t = \sum_{\tau=0}^{15} \xi_O(\tau|\mu_{\xi_O}, \sigma_{\xi_O}) I_{t-\tau} \tag{6}$$
$$D_t = \alpha \sum_{\tau=0}^{15} \xi_D(\tau|\mu_{\xi_D}, \sigma_{\xi_D}) O_{t-\tau} \tag{7}$$
$$C_t \sim \text{NB}\left(\omega_{(t \mod 7)} D_t, \phi\right) \tag{8}$$

Where,

$$w \sim \mathcal{G}(\mu_w, \sigma_w) \tag{9}$$
$$\xi_O \sim \mathcal{LN}(\mu_{\xi_O}, \sigma_{\xi_O}) \tag{10}$$
$$\xi_D \sim \mathcal{LN}(\mu_{\xi_D}, \sigma_{\xi_D}) \tag{11}$$

This model used the following priors for cases,

$$
\begin{aligned}
R_0 &\sim \mathcal{LN}(0.079, 0.18) && (12)\\
\mu_w &\sim \mathcal{N}(3.6, 0.7) && (13)\\
\sigma_w &\sim \mathcal{N}(3.1, 0.8) && (14)\\
\mu_{\xi_O} &\sim \mathcal{N}(1.62, 0.064) && (15)\\
\sigma_{\xi_O} &\sim \mathcal{N}(0.418, 0.069) && (16)\\
\mu_{\xi_D} &\sim \mathcal{N}(0.614, 0.066) && (17)\\
\sigma_{\xi_D} &\sim \mathcal{N}(1.51, 0.048) && (18)\\
\alpha &\sim \mathcal{N}(0.25, 0.05) && (19)\\
\frac{\omega}{7} &\sim \text{Dirichlet}(1, 1, 1, 1, 1, 1, 1) && (20)\\
\phi &\sim \frac{1}{\sqrt{\mathcal{N}(0, 1)}} && (21)
\end{aligned}
$$

and updated the reporting process as follows when forecasting deaths,

$$
\begin{aligned}
\mu_{\xi_D} &\sim \mathcal{N}(2.29, 0.076) && (22)\\
\sigma_{\xi_D} &\sim \mathcal{N}(0.76, 0.055) && (23)\\
\alpha &\sim \mathcal{N}(0.005, 0.0025) && (24)
\end{aligned}
$$

α, μ, σ, and ϕ were truncated to be greater than 0 and with ξ, and w normalised to sum to 1.

The prior for the generation time was sourced from (Ganyani et al. 2020) but refit using a log-normal incubation period with a mean of 5.2 days (SD 1.1) and SD of 1.52 days (SD 1.1) with this incubation period also being used as a prior (Lauer et al. 2020) for ξ_O. This resulted in a gamma-distributed generation time with mean 3.6 days (standard deviation (SD) 0.7), and SD of 3.1 days (SD 0.8) for all estimates. We estimated the delay between symptom onset and case report or death required to convolve latent infections to observations by fitting an integer adjusted log-normal distribution to 10 subsampled bootstraps of a public linelist for cases in Germany from April 2020 to June 2020 with each bootstrap using 1% or 1769 samples of the available data (Xu et al. 2020; Abbott, Sherratt, et al. 2020) and combining the posteriors for the mean and standard deviation of the log-normal distribution (Abbott, Hellewell, et al. 2020; epiforecasts.io/covid 2020; "Evaluating the Use of the Reproduction Number as an Epidemiological Tool, Using Spatio-Temporal Trends of the Covid-19 Outbreak in England | medRxiv" n.d.; Stan Development Team 2020).

GP_t is an approximate Hilbert space Gaussian process as defined in (Riutort-Mayol et al. 2020) using a Matern 3/2 kernel using a boundary factor of 1.5 and 17 basis functions (20% of the number of days used in fitting). The length scale of the Gaussian process was given a log-normal prior with a mean of 21 days, and a standard deviation of 7 days truncated to be greater than 3 days and less than 60 days. The magnitude of the Gaussian process was assumed to be normally distributed centred at 0 with a standard deviation of 0.1.

From the forecast time horizon (T) and onwards the last value of the Gaussian process was used (hence R_t was assumed to be fixed) and latent infections were adjusted to account for the proportion of the population that was susceptible to infection as follows,

$$
I_t = (N - I^c_{t-1}) \left(1 - \exp\left(\frac{-I'_t}{N - I^c_T}\right)\right), \tag{25}
$$

where $I^c_t = \sum_{s<t} I_s$ are cumulative infections by $t-1$ and I'_t are the unadjusted infections defined above. This adjustment is based on that implemented in the `epidemia` R package (Scott et al. 2020; Bhatt et al., n.d.).

0.1.1.1 Convolution model

The convolution model shares the same observation model as the renewal model but rather than assuming that an observation is predicted by itself using the renewal equation instead assumes that it is predicted entirely by another observation after some parametric delay. It can be defined mathematically as follows,

$$D_t \sim \text{NB}\left(\omega_{(t \mod 7)} \alpha \sum_{\tau=0}^{30} \xi(\tau|\mu,\sigma) C_{t-\tau}, \phi\right) \tag{26}$$

with the following priors,

$$\frac{\omega}{7} \sim \text{Dirichlet}(1,1,1,1,1,1,1) \tag{27}$$

$$\alpha \sim \mathcal{N}(0.01, 0.02) \tag{28}$$

$$\xi \sim \mathcal{LN}(\mu, \sigma) \tag{29}$$

$$\mu \sim \mathcal{N}(2.5, 0.5) \tag{30}$$

$$\sigma \sim \mathcal{N}(0.47, 0.2) \tag{31}$$

$$\phi \sim \frac{1}{\sqrt{\mathcal{N}(0,1)}} \tag{32}$$

with α, μ, σ, and ϕ truncated to be greater than 0 and with ξ normalised such that $\sum_{\tau=0}^{30} \xi(\tau|\mu,\sigma) = 1$.

0.1.2 Model fitting

Both models were implemented using the `EpiNow2` R package (version 1.3.3) (Abbott, Hellewell, et al. 2020). Each forecast target was fitted independently for each model using Markov-chain Monte Carlo (MCMC) in stan (Stan Development Team 2020). A minimum of 4 chains were used with a warmup of 250 samples for the renewal equation-based model and 1000 samples for the convolution model. 2000 samples total post warmup were used for the renewal equation model and 4000 samples for the convolution model. Different settings were chosen for each model to optimise compute time contingent on convergence. Convergence was assessed using the R hat diagnostic (Stan Development Team 2020). For the convolution model forecast the case forecast from the renewal equation model was used in place of observed cases beyond the forecast horizon using 1000 posterior samples. 12 weeks of data was used for both models though only 3 weeks of data were included in the likelihood for the convolution model.

Abbott, Sam, Joel Hellewell, Joe Hickson, James Munday, Katelyn Gostic, Peter Ellis, Katharine Sherratt, et al. 2020. "EpiNow2: Estimate Real-Time Case Counts and Time-Varying Epidemiological Parameters." - - (-): –. https://doi.org/10.5281/zenodo.3957489.

Abbott, Sam, Katharine Sherratt, Jonnie Bevan, Hamish Gibbs, Joel Hellewell, James Munday, Patrick Barks, Paul Campbell, Flavio Finger, and Sebastian Funk. 2020. "Covidregionaldata: Subnational Data for the Covid-19 Outbreak." - - (-): –. https://doi.org/10.5281/zenodo.3957539.

Bhatt, Samir, Neil Ferguson, Seth Flaxman, Axel Gandy, Swapnil Mishra, and James A Scott. n.d. "Semi-Mechanistic Bayesian Modeling of COVID-19 with Renewal Processes," 14.

epiforecasts.io/covid. 2020. "Covid-19: Temporal Variation in Transmission During the COVID-19 Outbreak." Covid-19. 2020. https://epiforecasts.io/covid/.

"Evaluating the Use of the Reproduction Number as an Epidemiological Tool, Using Spatio-Temporal Trends of the Covid-19 Outbreak in England | medRxiv." n.d. Accessed May 30, 2021. https://www.medrxiv.org/content/10.1101/2020.10.18.20214585v1.

Ganyani, Tapiwa, Cecile Kremer, Dongxuan Chen, Andrea Torneri, Christel Faes, Jacco Wallinga, and Niel Hens. 2020. "Estimating the Generation Interval for Coronavirus Disease (COVID-19) Based on Symptom Onset Data, March 2020." *Eurosurveillance* 25 (17).

Lauer, Stephen A, Kyra H Grantz, Qifang Bi, Forrest K Jones, Qulu Zheng, Hannah R Meredith, Andrew S Azman, Nicholas G Reich, and Justin Lessler. 2020. "The Incubation Period of Coronavirus Disease 2019 (COVID-19) from Publicly Reported Confirmed Cases: Estimation and Application." *Annals of Internal Medicine* 172 (9): 577–82.

Riutort-Mayol, Gabriel, Paul-Christian Bürkner, Michael R. Andersen, Arno Solin, and Aki Vehtari. 2020. "Practical Hilbert Space Approximate Bayesian Gaussian Processes for Probabilistic Programming." https://arxiv.org/abs/2004.11408.

Scott, James A., Axel Gandy, Swapnil Mishra, Juliette Unwin, Seth Flaxman, and Samir Bhatt. 2020. "Epidemia: Modeling of Epidemics Using Hierarchical Bayesian Models." https://imperialcollegelondon.github.io/epidemia/.

Stan Development Team. 2020. "RStan: The r Interface to Stan." http://mc-stan.org/.

Xu, Bo, Bernardo Gutierrez, Sarah Hill, Samuel Scarpino, Alyssa Loskill, Jessie Wu, Kara Sewalk, et al. 2020. "Epidemiological Data from the nCoV-2019 Outbreak: Early Descriptions from Publicly Available Data." http://virological.org/t/epidemiological-data-from-the-ncov-2019-outbreak-early-descriptions-from-publicly-available-data/337.
