# Supplementary material for: Comparing human and model-based forecasts of COVID-19 in Germany and Poland
Source: PLoS Comput Biol. 2022 Sep 19;18(9):e1010405. doi: 10.1371/journal.pcbi.1010405 (PMC9534421; doi:10.1371/journal.pcbi.1010405)
Supplement: S1 Table — (PDF) [file pcbi.1010405.s002.pdf]

Metric	Explanation
WIS (Weighted) interval score	The weighted interval score (smaller values are better) is a proper scoring rule for quantile forecasts. It converges to the continuos ranked probability score (which itself is a generalisation of the absolute error to probabilistic forecasts) for an increasing number of intervals. The score can be decomposed into a dispersion (uncertainty) component and penalties for over- and underprediction. For a single interval, the score is computed as $$IS_\alpha(F,y) = (u-l) + \frac{2}{\alpha} \cdot (l-y) \cdot 1(y \leq l) + \frac{2}{\alpha} \cdot (y-u) \cdot 1(y \geq u),$$ where 1() is the indicator function, y is the true value, and l and u are the $\frac{\alpha}{2}$ and $1 - \frac{\alpha}{2}$ quantiles of the predictive distribution F, i.e. the lower and upper bound of a single prediction interval. For a set of K prediction intervals and the median m, the score is computed as a weighted sum, $$WIS = \frac{1}{K+0.5} \cdot \left(w_0 \cdot \lvert y - m \rvert + \sum_{k=1}^{K} w_k \cdot IS_\alpha(F,y) \right),$$ where w_k is a weight for every interval. Usually, $w_k = \frac{\alpha_k}{2}$ and $w_0 = 0.5$.<br>Its proximity to the absolute error means that when averaging across multiple targets (e.g. different weeks), it will be dominated by targets with higher absolute values.
Interval coverage	Interval coverage is a measure of marginal calibration and indicates the proportion of observed values that fall in a given prediction interval range. Nominal coverage represents the percentage of observed values that should ideally be covered (e.g. we would like a 50 percent prediction interval to cover on average 50 percent of the observations), while empirical coverage is the actual percentage of observations covered by a certain prediction interval.
Bias	(Relative) bias is a measure of the general tendency of a forecaster to over- or underpredict. Values are between -1 and 1 and 0 ideally. For continuous forecasts, bias is given as $$B(F,y) = 1 - 2 \cdot (F(y)),$$ where F is the CDF of the predictive distribution and y is the observed value.<br>For quantile forecasts, $F(y)$ is replaced by a quantile rank. The appropriate quantile rank is determined by whether the median forecast is below or above the true value. We then take the innermost quantile rank for which the quantile is still larger (under-prediction) or smaller (over-prediction) than the observed value.<br>In contrast to the over- and underprediction penalties of the interval score it is bound between 0 and 1 and represents a general tendency of forecasts to be biased rather than the absolute amount of over- and underprediction. It is therefore a more robust measurement.
