# Supplementary material for: Comparing human and model-based forecasts of COVID-19 in Germany and Poland
Source: PLoS Comput Biol. 2022 Sep 19;18(9):e1010405. doi: 10.1371/journal.pcbi.1010405 (PMC9534421; doi:10.1371/journal.pcbi.1010405)
Supplement: S4 Table — Scores are cut to three significant digits and rounded. Note that scores for cases (which include the whole period from October 12th 2020 until March 1st 2021) and deaths (which include only forecasts from the 21st of December 2020 on) are computed on different subsets. Numbers in brackets show the metrics relative to the Hub ensemble (i.e. the median ensemble of all other models submitted to the German and Polish Forecast Hub, excluding our contributions). WIS is the mean weighted interval score (lower values are better), WIS—sd is the standard deviation of all scores achieved by a model. Dispersion, over-prediction and under-prediction together sum up to the weighted interval score. Bias (between -1 and 1, 0 is ideal) represents the general average tendency of a model to over- or underpredict. 50% and 90%-coverage are the percentage of observed values that fell within the 50% and 90% prediction intervals of a model. (PDF) [file pcbi.1010405.s005.pdf]

	Model	WIS	WIS - sd	dispersion	Underpred.	Overpred.	Bias	Abs. error	50%-Cov.	90%-Cov.
**Cases**										
1 wk ahead	Hub-ensemble	8770 (1)	11700 (1)	3670 (1)	1230 (1)	3870 (1)	-0.04	12700 (1)	0.57	0.81
	Hub-ensemble-realised	6970 (0.79)	8260 (0.71)	3060 (0.83)	943 (0.77)	2970 (0.77)	0.04	10800 (0.85)	0.55	0.83
	Hub-ensemble-with-crowd	7820 (0.89)	9630 (0.82)	3270 (0.89)	1210 (0.98)	3330 (0.86)	-0.02	12000 (0.94)	0.48	0.81
	Hub-ensemble-with-renewal	7960 (0.91)	10300 (0.88)	3190 (0.87)	1020 (0.83)	3760 (0.97)	0.04	12100 (0.95)	0.57	0.83
2 wk ahead	Hub-ensemble	18300 (1)	21900 (1)	6140 (1)	3800 (1)	8410 (1)	-0.03	26800 (1)	0.43	0.64
	Hub-ensemble-realised	16400 (0.9)	19600 (0.89)	5350 (0.87)	3290 (0.87)	7730 (0.92)	0.02	24200 (0.9)	0.43	0.69
	Hub-ensemble-with-crowd	16900 (0.92)	19600 (0.89)	5230 (0.85)	4310 (1.13)	7370 (0.88)	0.00	24600 (0.92)	0.38	0.64
	Hub-ensemble-with-renewal	17500 (0.96)	21400 (0.98)	5830 (0.95)	2880 (0.76)	8770 (1.04)	0.00	25500 (0.95)	0.45	0.71
**Deaths**										
1 wk ahead	Hub-ensemble	248 (1)	338 (1)	92.2 (1)	115 (1)	41.6 (1)	-0.04	334 (1)	0.62	0.92
	Hub-ensemble-realised	235 (0.95)	332 (0.98)	88.6 (0.96)	90.4 (0.79)	55.5 (1.33)	-0.01	323 (0.97)	0.62	0.88
	Hub-ensemble-with-all	234 (0.94)	331 (0.98)	85.2 (0.92)	98.1 (0.85)	50.2 (1.21)	-0.05	329 (0.99)	0.62	0.92
	Hub-ensemble-with-convolution	234 (0.94)	329 (0.97)	90.7 (0.98)	118 (1.03)	25.3 (0.61)	-0.08	333 (1)	0.62	0.92
	Hub-ensemble-with-crowd	239 (0.96)	337 (1)	85.2 (0.92)	99.6 (0.87)	54.2 (1.3)	-0.03	322 (0.96)	0.62	0.92
	Hub-ensemble-with-renewal	246 (0.99)	342 (1.01)	91.5 (0.99)	106 (0.92)	48.6 (1.17)	-0.06	342 (1.02)	0.67	0.92
2 wk ahead	Hub-ensemble	292 (1)	385 (1)	132 (1)	108 (1)	51.9 (1)	0.01	429 (1)	0.62	0.96
	Hub-ensemble-realised	296 (1.01)	398 (1.03)	125 (0.95)	91 (0.84)	80.2 (1.55)	0.05	486 (1.13)	0.58	0.92
	Hub-ensemble-with-all	303 (1.04)	423 (1.1)	115 (0.87)	122 (1.13)	66.1 (1.27)	0.00	483 (1.13)	0.62	0.88
	Hub-ensemble-with-convolution	270 (0.92)	385 (1)	121 (0.92)	119 (1.1)	29.9 (0.58)	-0.04	403 (0.94)	0.58	0.96
	Hub-ensemble-with-crowd	303 (1.04)	392 (1.02)	122 (0.92)	106 (0.98)	74.6 (1.44)	0.03	499 (1.16)	0.58	0.92
	Hub-ensemble-with-renewal	296 (1.01)	397 (1.03)	128 (0.97)	97.1 (0.9)	71.2 (1.37)	-0.01	462 (1.08)	0.67	0.92
