# Supplementary material for: Comparing human and model-based forecasts of COVID-19 in Germany and Poland
Source: PLoS Comput Biol. 2022 Sep 19;18(9):e1010405. doi: 10.1371/journal.pcbi.1010405 (PMC9534421; doi:10.1371/journal.pcbi.1010405)
Supplement: S6 Table — Scores are cut to three significant digits and rounded. Note that scores for cases (which include the whole period from October 12th 2020 until March 1st 2021) and deaths (which include only forecasts from the 21st of December 2020 on) are computed on different subsets. Numbers in brackets show the metrics relative to the Hub mean ensemble (i.e. the mean ensemble of all other models submitted to the German and Polish Forecast Hub, excluding our contributions). WIS is the mean weighted interval score (lower values are better), WIS—sd is the standard deviation of all scores achieved by a model. Dispersion, over-prediction and under-prediction together sum up to the weighted interval score. Bias (between -1 and 1, 0 is ideal) represents the general average tendency of a model to over- or underpredict. 50% and 90%-coverage are the percentage of observed values that fell within the 50% and 90% prediction intervals of a model. (PDF) [file pcbi.1010405.s007.pdf]

	Model	WIS	WIS - sd	dispersion	Underpred.	Overpred.	Bias	Abs. error	50%-Cov.	90%-Cov.
**Cases**										
1 wk ahead	Hub-ensemble-mean	8680 (1)	10300 (1)	3700 (1)	1460 (1)	3520 (1)	-0.02	13400 (1)	0.50	0.86
	Hub-ensemble-realised-mean	7600 (0.88)	8770 (0.85)	3360 (0.91)	1090 (0.75)	3140 (0.89)	0.01	11900 (0.89)	0.52	0.90
	Hub-ensemble-with-crowd-mean	8050 (0.93)	9070 (0.88)	3520 (0.95)	1410 (0.97)	3120 (0.89)	-0.02	12600 (0.94)	0.48	0.88
	Hub-ensemble-with-renewal-mean	8090 (0.93)	9780 (0.95)	3490 (0.94)	1110 (0.76)	3490 (0.99)	0.02	12700 (0.95)	0.57	0.88
2 wk ahead	Hub-ensemble-mean	19000 (1)	22100 (1)	5960 (1)	3690 (1)	9340 (1)	0.02	28800 (1)	0.33	0.79
	Hub-ensemble-realised-mean	17100 (0.9)	20600 (0.93)	5550 (0.93)	2850 (0.77)	8660 (0.93)	0.05	26000 (0.9)	0.38	0.76
	Hub-ensemble-with-crowd-mean	17600 (0.93)	20000 (0.9)	5540 (0.93)	3790 (1.03)	8230 (0.88)	0.01	26800 (0.93)	0.36	0.76
	Hub-ensemble-with-renewal-mean	18300 (0.96)	22600 (1.02)	5910 (0.99)	2640 (0.72)	9720 (1.04)	0.06	27700 (0.96)	0.38	0.76
**Deaths**										
1 wk ahead	Hub-ensemble-mean	229 (1)	292 (1)	101 (1)	90.4 (1)	36.7 (1)	-0.07	315 (1)	0.71	0.92
	Hub-ensemble-realised-mean	219 (0.96)	289 (0.99)	96.8 (0.96)	79.8 (0.88)	42.6 (1.16)	-0.04	297 (0.94)	0.71	0.88
	Hub-ensemble-with-all-mean	217 (0.95)	287 (0.98)	95.3 (0.94)	83.1 (0.92)	38.7 (1.05)	-0.07	300 (0.95)	0.67	0.88
	Hub-ensemble-with-convolution-mean	225 (0.98)	292 (1)	98.7 (0.98)	94.2 (1.04)	32 (0.87)	-0.09	314 (1)	0.71	0.92
	Hub-ensemble-with-crowd-mean	222 (0.97)	289 (0.99)	98 (0.97)	84.1 (0.93)	39.6 (1.08)	-0.04	295 (0.94)	0.71	0.88
	Hub-ensemble-with-renewal-mean	225 (0.98)	290 (0.99)	99.7 (0.99)	84.7 (0.94)	40.5 (1.1)	-0.05	314 (1)	0.71	0.88
2 wk ahead	Hub-ensemble-mean	256 (1)	306 (1)	138 (1)	64.5 (1)	53.2 (1)	0.04	374 (1)	0.67	0.96
	Hub-ensemble-realised-mean	270 (1.05)	338 (1.1)	136 (0.99)	65.2 (1.01)	68.1 (1.28)	0.06	413 (1.1)	0.75	0.92
	Hub-ensemble-with-all-mean	268 (1.05)	346 (1.13)	133 (0.96)	78.7 (1.22)	57.1 (1.07)	0.05	408 (1.09)	0.75	0.96
	Hub-ensemble-with-convolution-mean	259 (1.01)	322 (1.05)	133 (0.96)	81.7 (1.27)	44.4 (0.83)	0.03	380 (1.02)	0.67	0.96
	Hub-ensemble-with-crowd-mean	264 (1.03)	315 (1.03)	133 (0.96)	70.1 (1.09)	60 (1.13)	0.06	404 (1.08)	0.71	0.96
	Hub-ensemble-with-renewal-mean	264 (1.03)	332 (1.08)	141 (1.02)	60.1 (0.93)	63.1 (1.19)	0.06	390 (1.04)	0.79	0.92
