# Supplementary material for: Comparing human and model-based forecasts of COVID-19 in Germany and Poland
Source: PLoS Comput Biol. 2022 Sep 19;18(9):e1010405. doi: 10.1371/journal.pcbi.1010405 (PMC9534421; doi:10.1371/journal.pcbi.1010405)
Supplement: S7 Table — Scores are cut to three significant digits and rounded. Note that scores for cases (which include the whole period from October 12th 2020 until March 1st 2021) and deaths (which include only forecasts from the 21st of December 2020 on) are computed on different subsets. Numbers in brackets show the metrics relative to the Hub mean ensemble (i.e. the mean ensemble of all other models submitted to the German and Polish Forecast Hub, excluding our contributions). WIS is the mean weighted interval score (lower values are better), WIS—sd is the standard deviation of all scores achieved by a model. Dispersion, over-prediction and under-prediction together sum up to the weighted interval score. Bias (between -1 and 1, 0 is ideal) represents the general average tendency of a model to over- or underpredict. 50% and 90%-coverage are the percentage of observed values that fell within the 50% and 90% prediction intervals of a model. (PDF) [file pcbi.1010405.s008.pdf]

	Model	WIS	WIS - sd	dispersion	Underpred.	Overpred.	Bias	Abs. error	50%-Cov.	90%-Cov.
**Cases**										
3 wk ahead	Hub-ensemble-mean	35600 (1)	42100 (1)	9340 (1)	7050 (1)	19200 (1)	0.03	51200 (1)	0.26	0.62
	Hub-ensemble-realised-mean	32100 (0.9)	40500 (0.96)	8830 (0.95)	4920 (0.7)	18300 (0.95)	0.07	47200 (0.92)	0.29	0.64
	Hub-ensemble-with-crowd-mean	32200 (0.9)	36700 (0.87)	8430 (0.9)	7190 (1.02)	16500 (0.86)	0.04	46900 (0.92)	0.24	0.64
	Hub-ensemble-with-renewal-mean	35200 (0.99)	46000 (1.09)	9630 (1.03)	4600 (0.65)	20900 (1.09)	0.08	51000 (1)	0.38	0.67
4 wk ahead	Hub-ensemble-mean	60300 (1)	79300 (1)	15700 (1)	10400 (1)	34100 (1)	0.04	78600 (1)	0.29	0.57
	Hub-ensemble-realised-mean	55000 (0.91)	77100 (0.97)	14600 (0.93)	6620 (0.64)	33800 (0.99)	0.11	75200 (0.96)	0.33	0.64
	Hub-ensemble-with-crowd-mean	53400 (0.89)	66600 (0.84)	13700 (0.87)	10600 (1.02)	29200 (0.86)	0.06	70400 (0.9)	0.26	0.60
	Hub-ensemble-with-renewal-mean	61700 (1.02)	89800 (1.13)	16400 (1.04)	6400 (0.62)	38900 (1.14)	0.12	82900 (1.05)	0.31	0.64
**Deaths**										
3 wk ahead	Hub-ensemble-mean	289 (1)	293 (1)	178 (1)	45.9 (1)	65.7 (1)	0.01	443 (1)	0.58	1.00
	Hub-ensemble-realised-mean	310 (1.07)	348 (1.19)	182 (1.02)	42 (0.92)	86.5 (1.32)	0.08	502 (1.13)	0.58	1.00
	Hub-ensemble-with-all-mean	315 (1.09)	339 (1.16)	178 (1)	62.2 (1.36)	74 (1.13)	0.07	507 (1.14)	0.62	1.00
	Hub-ensemble-with-convolution-mean	297 (1.03)	292 (1)	174 (0.98)	67.7 (1.47)	55 (0.84)	0.01	452 (1.02)	0.67	1.00
	Hub-ensemble-with-crowd-mean	294 (1.02)	299 (1.02)	172 (0.97)	48 (1.05)	74.2 (1.13)	0.03	476 (1.07)	0.58	1.00
	Hub-ensemble-with-renewal-mean	310 (1.07)	349 (1.19)	189 (1.06)	39.4 (0.86)	81.9 (1.25)	0.05	482 (1.09)	0.62	1.00
4 wk ahead	Hub-ensemble-mean	437 (1)	568 (1)	232 (1)	72 (1)	134 (1)	0.00	702 (1)	0.62	1.00
	Hub-ensemble-realised-mean	445 (1.02)	598 (1.05)	237 (1.02)	56.4 (0.78)	152 (1.13)	0.06	707 (1.01)	0.58	1.00
	Hub-ensemble-with-all-mean	421 (0.96)	520 (0.92)	239 (1.03)	49.9 (0.69)	132 (0.99)	0.05	678 (0.97)	0.58	1.00
	Hub-ensemble-with-convolution-mean	398 (0.91)	465 (0.82)	235 (1.01)	55.6 (0.77)	107 (0.8)	0.00	628 (0.89)	0.67	1.00
	Hub-ensemble-with-crowd-mean	418 (0.96)	533 (0.94)	222 (0.96)	66.8 (0.93)	129 (0.96)	0.03	662 (0.94)	0.58	1.00
	Hub-ensemble-with-renewal-mean	467 (1.07)	636 (1.12)	248 (1.07)	61 (0.85)	158 (1.18)	0.05	755 (1.08)	0.67	1.00
