# Supplementary material for: Comparing human and model-based forecasts of COVID-19 in Germany and Poland
Source: PLoS Comput Biol. 2022 Sep 19;18(9):e1010405. doi: 10.1371/journal.pcbi.1010405 (PMC9534421; doi:10.1371/journal.pcbi.1010405)
Supplement: S8 Table — Scores are cut to three significant digits and rounded. In the original analysis, cases and deaths were scored on different periods, as the convolution model was only added later. This table shows performance of all models restricted to the period from December 14 2020 until March 1st 2021 where all models were available. Numbers in brackets show the metrics relative to the Hub ensemble (i.e. the median ensemble of all other models submitted to the German and Polish Forecast Hub, excluding our contributions). WIS is the mean weighted interval score (lower values are better), WIS—sd is the standard deviation of all scores achieved by a model. Dispersion, over-prediction and under-prediction together sum up to the weighted interval score. Bias (between -1 and 1, 0 is ideal) represents the general average tendency of a model to over- or underpredict. 50% and 90%-coverage are the percentage of observed values that fell within the 50% and 90% prediction intervals of a model. (PDF) [file pcbi.1010405.s009.pdf]

	Model	WIS	WIS - sd	dispersion	Underpred.	Overpred.	Bias	Abs. error	50%-Cov.	90%-Cov.
**Cases**										
1 wk ahead	Crowd forecast	4980 (0.74)	5730 (0.64)	2070 (0.6)	728 (0.74)	2190 (0.94)	0.09	7810 (0.82)	0.54	0.88
	Hub-ensemble	6730 (1)	8960 (1)	3430 (1)	978 (1)	2330 (1)	-0.09	9550 (1)	0.62	0.92
	Renewal	9640 (1.43)	13300 (1.48)	1970 (0.57)	4170 (4.26)	3500 (1.5)	0.09	12700 (1.33)	0.46	0.71
2 wk ahead	Crowd forecast	10700 (0.99)	13800 (1.1)	2880 (0.58)	2350 (0.85)	5430 (1.79)	0.08	15400 (1.07)	0.46	0.62
	Hub-ensemble	10800 (1)	12500 (1)	4940 (1)	2780 (1)	3030 (1)	-0.13	14400 (1)	0.54	0.75
	Renewal	25000 (2.31)	34000 (2.72)	4780 (0.97)	8710 (3.13)	11500 (3.8)	0.05	32000 (2.22)	0.50	0.67
**Deaths**										
1 wk ahead	Convolution	255 (1.03)	343 (1.01)	82 (0.89)	142 (1.23)	31.1 (0.75)	-0.18	399 (1.19)	0.42	0.79
	Crowd forecast	265 (1.07)	317 (0.94)	78.2 (0.85)	82 (0.71)	105 (2.52)	0.08	402 (1.2)	0.38	0.79
	Hub-ensemble	248 (1)	338 (1)	92.2 (1)	115 (1)	41.6 (1)	-0.04	334 (1)	0.62	0.92
	Renewal	298 (1.2)	403 (1.19)	87 (0.94)	107 (0.93)	105 (2.52)	-0.07	413 (1.24)	0.50	0.79
2 wk ahead	Convolution	357 (1.22)	573 (1.49)	104 (0.79)	204 (1.89)	48.8 (0.94)	-0.10	565 (1.32)	0.33	0.79
	Crowd forecast	368 (1.26)	442 (1.15)	107 (0.81)	102 (0.94)	160 (3.08)	0.14	576 (1.34)	0.38	0.75
	Hub-ensemble	292 (1)	385 (1)	132 (1)	108 (1)	51.9 (1)	0.01	429 (1)	0.62	0.96
	Renewal	524 (1.79)	671 (1.74)	155 (1.17)	133 (1.23)	236 (4.55)	-0.02	750 (1.75)	0.50	0.71
