# Supplementary material for: Comparing human and model-based forecasts of COVID-19 in Germany and Poland
Source: PLoS Comput Biol. 2022 Sep 19;18(9):e1010405. doi: 10.1371/journal.pcbi.1010405 (PMC9534421; doi:10.1371/journal.pcbi.1010405)
Supplement: S9 Table — Scores are cut to three significant digits and rounded. In the original analysis, cases and deaths were scored on different periods, as the convolution model was only added later. This table shows performance of all models restricted to the period from December 14 2020 until March 1st 2021 where all models were available. Numbers in brackets show the metrics relative to the Hub ensemble (i.e. the median ensemble of all other models submitted to the German and Polish Forecast Hub, excluding our contributions). WIS is the mean weighted interval score (lower values are better), WIS—sd is the standard deviation of all scores achieved by a model. Dispersion, over-prediction and under-prediction together sum up to the weighted interval score. Bias (between -1 and 1, 0 is ideal) represents the general average tendency of a model to over- or underpredict. 50% and 90%-coverage are the percentage of observed values that fell within the 50% and 90% prediction intervals of a model. (PDF) [file pcbi.1010405.s010.pdf]

	Model	WIS	WIS - sd	dispersion	Underpred.	Overpred.	Bias	Abs. error	50%-Cov.	90%-Cov.
**Cases**										
3 wk ahead	Crowd forecast	17200 (1)	16000 (0.98)	3800 (0.63)	5660 (0.85)	7770 (1.74)	0.07	26800 (1.1)	0.08	0.58
	Hub-ensemble	17200 (1)	16300 (1)	6030 (1)	6670 (1)	4470 (1)	-0.16	24400 (1)	0.33	0.67
	Renewal	37700 (2.19)	55900 (3.43)	8840 (1.47)	10700 (1.6)	18100 (4.05)	-0.03	49800 (2.04)	0.33	0.58
4 wk ahead	Crowd forecast	26100 (0.95)	21000 (0.84)	4810 (0.7)	11300 (0.83)	10100 (1.43)	0.04	39400 (1.05)	0.00	0.46
	Hub-ensemble	27600 (1)	25000 (1)	6900 (1)	13600 (1)	7060 (1)	-0.19	37400 (1)	0.29	0.54
	Renewal	48900 (1.77)	77800 (3.11)	13800 (2)	11900 (0.88)	23200 (3.29)	-0.10	65500 (1.75)	0.38	0.58
**Deaths**										
3 wk ahead	Convolution	541 (1.7)	802 (2.45)	157 (0.91)	279 (3.01)	105 (1.91)	-0.04	747 (1.53)	0.54	0.75
	Crowd forecast	414 (1.3)	526 (1.6)	137 (0.8)	82 (0.88)	194 (3.52)	0.12	648 (1.33)	0.42	0.83
	Hub-ensemble	319 (1)	328 (1)	172 (1)	92.7 (1)	55.1 (1)	-0.03	488 (1)	0.54	0.96
	Renewal	724 (2.27)	916 (2.79)	249 (1.45)	158 (1.7)	317 (5.75)	-0.01	1040 (2.13)	0.46	0.83
4 wk ahead	Convolution	763 (1.8)	932 (2.1)	268 (1.26)	331 (2.63)	164 (1.91)	0.01	985 (1.46)	0.54	0.75
	Crowd forecast	498 (1.17)	633 (1.43)	168 (0.79)	83.6 (0.66)	246 (2.87)	0.14	756 (1.12)	0.38	0.79
	Hub-ensemble	424 (1)	443 (1)	212 (1)	126 (1)	85.7 (1)	-0.06	675 (1)	0.58	0.92
	Renewal	959 (2.26)	1210 (2.73)	337 (1.59)	200 (1.59)	421 (4.91)	-0.05	1350 (2)	0.50	0.79
