# Supplementary material for: Comparing human and model-based forecasts of COVID-19 in Germany and Poland
Source: PLoS Comput Biol. 2022 Sep 19;18(9):e1010405. doi: 10.1371/journal.pcbi.1010405 (PMC9534421; doi:10.1371/journal.pcbi.1010405)
Supplement: S10 Table — (PDF) [file pcbi.1010405.s011.pdf]

Name	Explanation
Hub-ensemble-realised	Official Forecast Hub median ensemble. Created by the Forecast Hub officially under the name 'KITCOVIDhub-median_ensemble' and used as the default ensemble. Included are our crowd forecasts as well as the renewal model (with one missed submission on December 28 2020, but not the convolution model which was deemed to similar to the renewal model.
Hub-ensemble-realised-mean	Official Forecast Hub mean ensemble. Created by the Forecast Hub officially under the name 'KITCOVIDhub-mean_ensemble'.
Hub-ensemble	Version of the official Hub median ensemble which excludes all our contributions.
Hub-ensemble-mean	Version of the official Hub mean ensemble which excludes all our contributions.
Hub-ensemble-with-renewal, Hub-ensemble-with-renewal-mean	Versions of the official Hub ensembles which of our contributions includes only the Renewal model.
Hub-ensemble-with-crowd, Hub-ensemble-with-crowd-mean	Versions of the official Hub ensembles which of our contributions includes only the Crowd forecast.
Hub-ensemble-with-convolution, Hub-ensemble-with-convolution-mean	Versions of the official Hub ensembles which of our contributions includes only the Convolution model (which originally was never included in any official Hub ensemble).
Hub-ensemble-with-all, Hub-ensemble-with-all-mean	Versions of the official Hub ensembles which includes all our contributions. For cases, this is identical to the official Hub ensembles, but for deaths the convolution model was added.
Crowd forecast	Submitted to the Forecast Hub as 'epiforecasts-EpiExpert'
Renewal model	Submitted to the Forecast Hub as 'epiforecasts-EpiNow2'
Convolution model	Submitted to the Forecast Hub as 'epiforecasts-EpiNow2_secondary'
