# Supplementary figures and images for: Comparing human and model-based forecasts of COVID-19 in Germany and Poland
Source: PLoS Comput Biol. 2022 Sep 19;18(9):e1010405. doi: 10.1371/journal.pcbi.1010405 (PMC9534421; doi:10.1371/journal.pcbi.1010405)

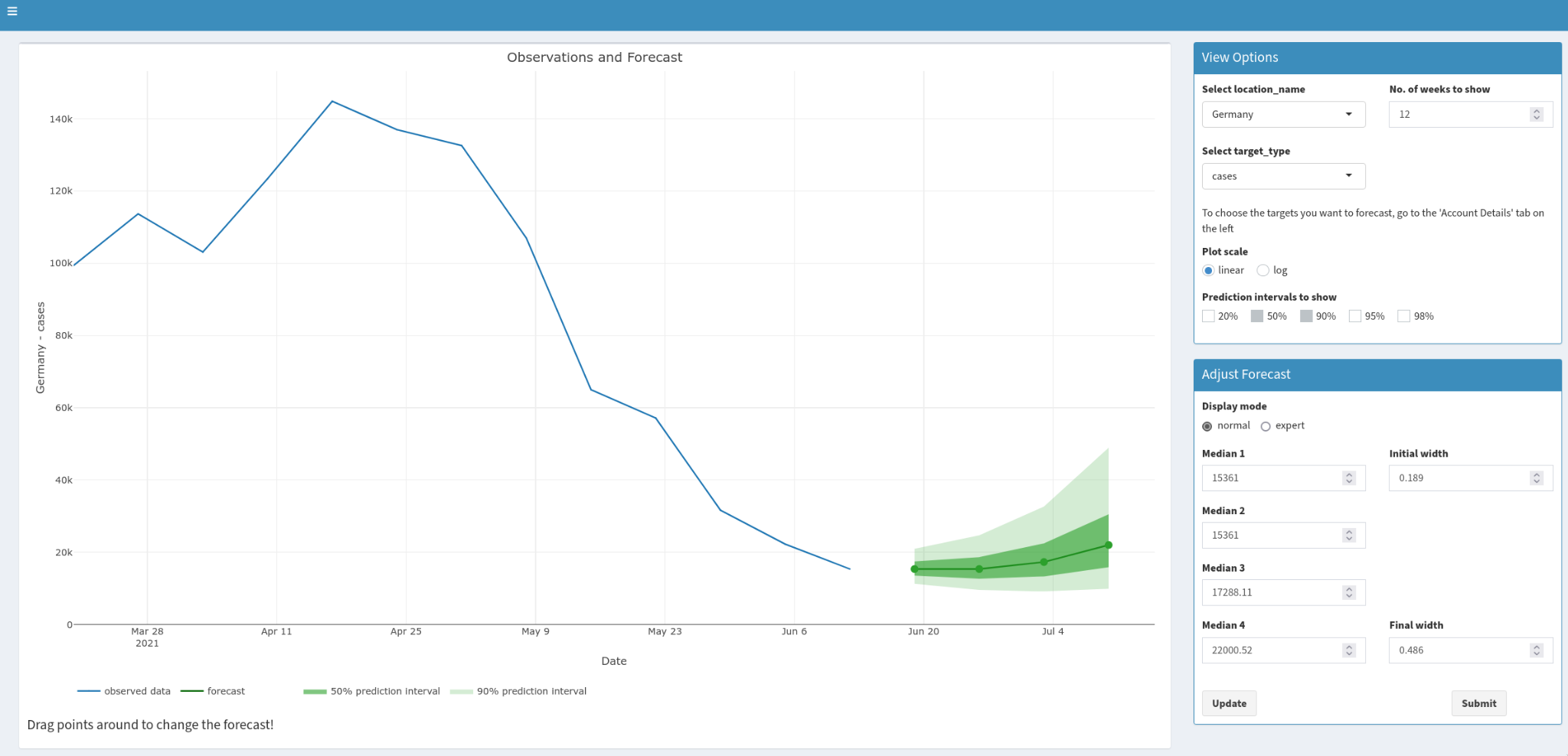

Supplement: S1 Fig — (TIF) [file pcbi.1010405.s012.tif]

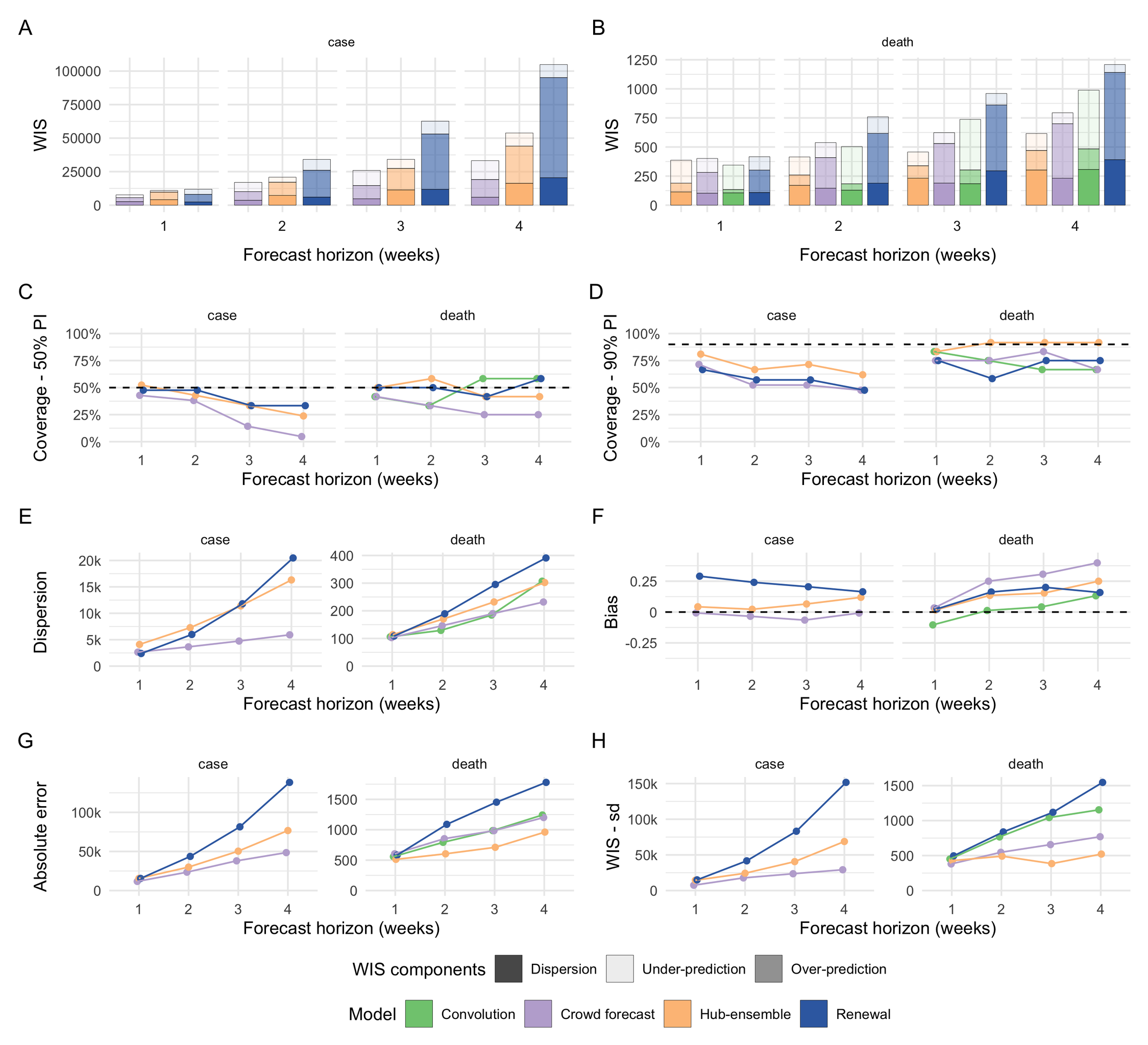

Supplement: S2 Fig — A, B: mean weighted interval score (WIS, lower indicates better performance) across horizons. WIS is decomposed into its components dispersion, over-prediction and under-prediction. C: Empirical coverage of the 50% prediction intervals (50% coverage is perfect). D: Empirical coverage of the 90% prediction intervals. E: Dispersion (same as in panel A, B). Higher values mean greater dispersion of the forecast and imply ceteris paribus a worse score. F: Bias, i.e. general (relative) tendency to over- or underpredict. Values are between -1 (complete under-prediction) and 1 (complete over-prediction) and 0 ideally. G: Absolute error of the median forecast (lower is better). H. Standard deviation of all WIS values for different horizons (TIF) [file pcbi.1010405.s013.tif]

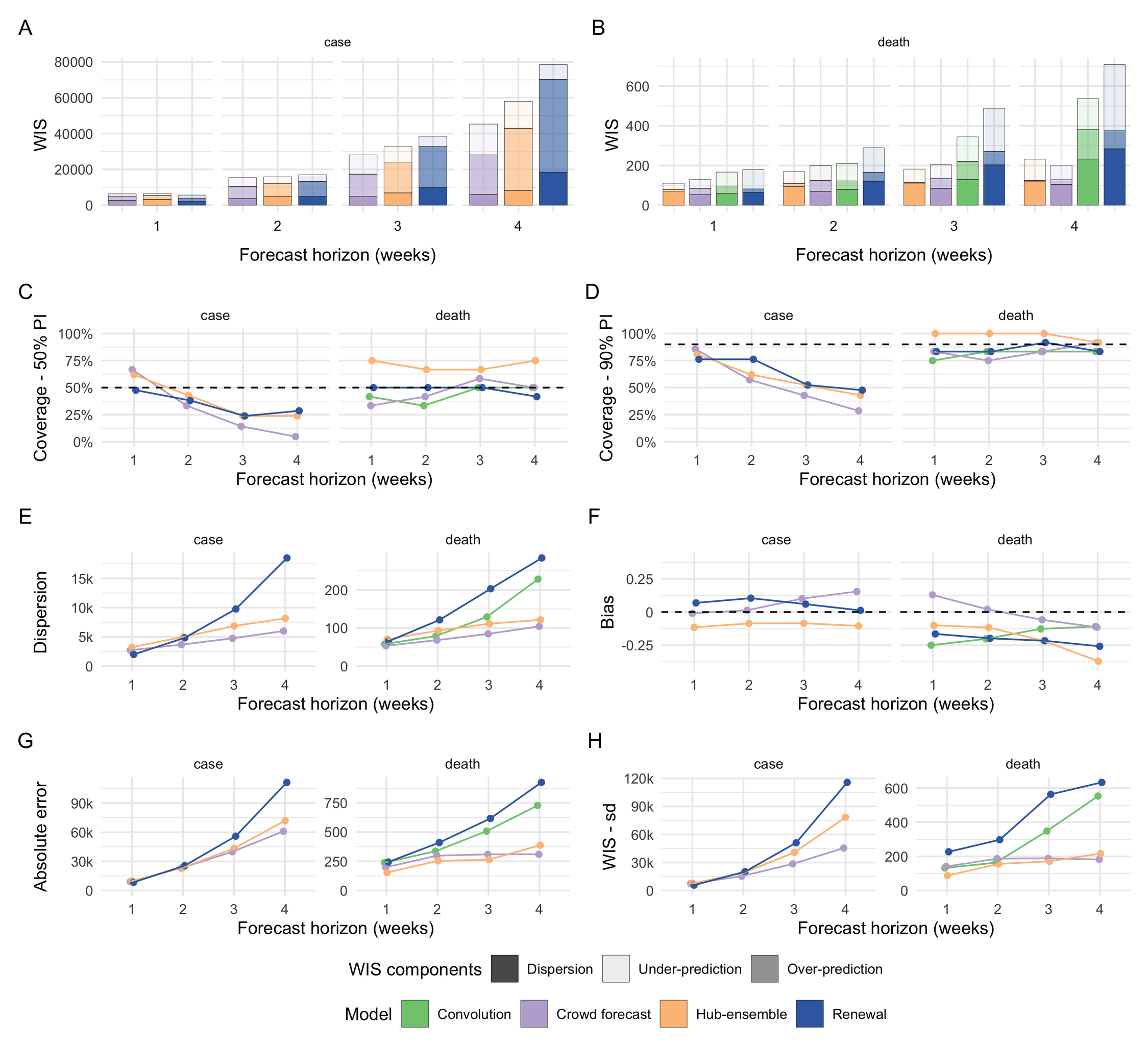

Supplement: S3 Fig — A, B: mean weighted interval score (WIS, lower indicates better performance) across horizons. WIS is decomposed into its components dispersion, over-prediction and under-prediction. C: Empirical coverage of the 50% prediction intervals (50% coverage is perfect). D: Empirical coverage of the 90% prediction intervals. E: Dispersion (same as in panel A, B). Higher values mean greater dispersion of the forecast and imply ceteris paribus a worse score. F: Bias, i.e. general (relative) tendency to over- or underpredict. Values are between -1 (complete under-prediction) and 1 (complete over-prediction) and 0 ideally. G: Absolute error of the median forecast (lower is better). H. Standard deviation of all WIS values for different horizons. (TIF) [file pcbi.1010405.s014.tif]

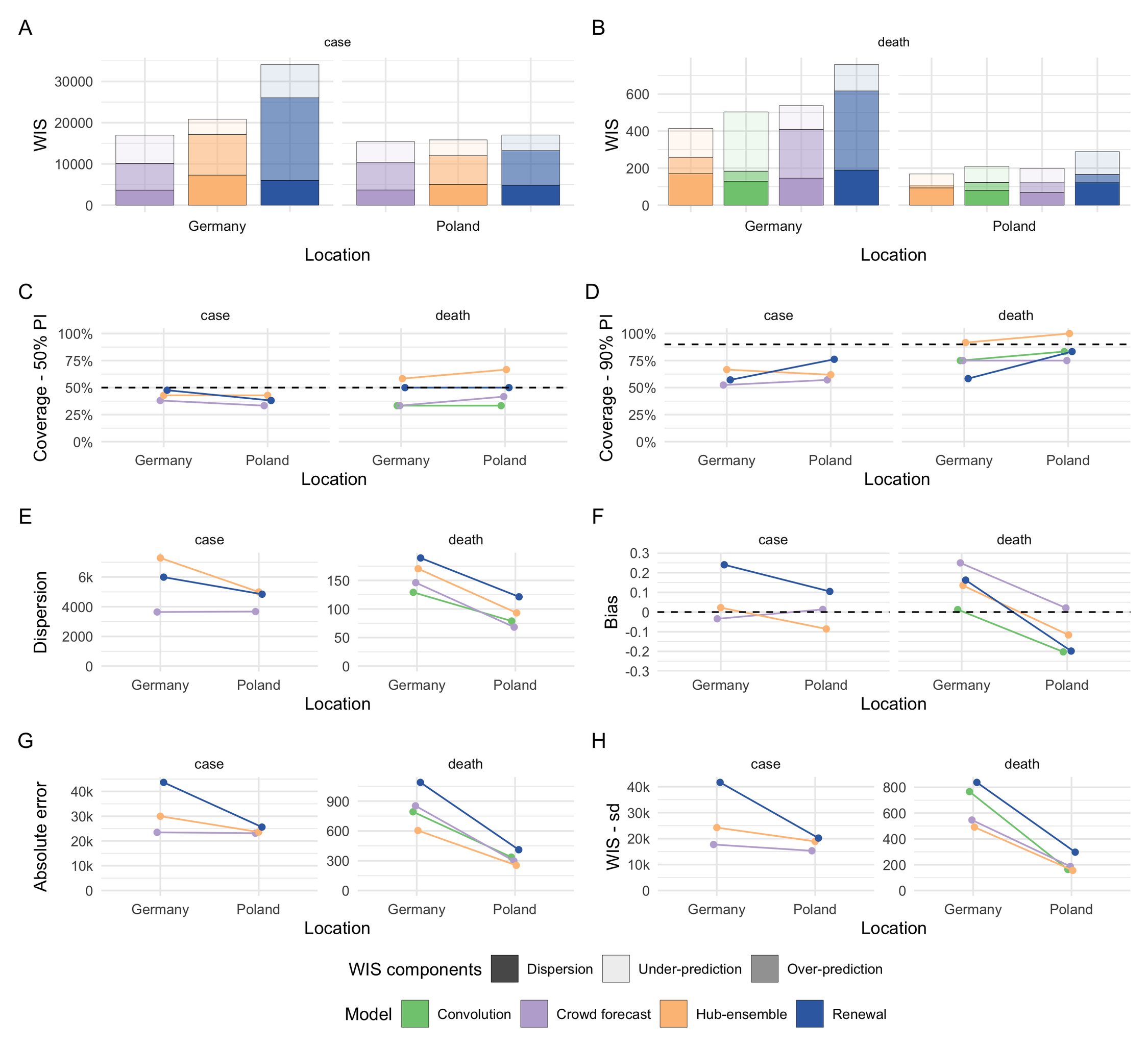

Supplement: S4 Fig — A, B: mean weighted interval score (WIS, lower indicates better performance) across horizons. WIS is decomposed into its components dispersion, over-prediction and under-prediction. C: Empirical coverage of the 50% prediction intervals (50% coverage is perfect). D: Empirical coverage of the 90% prediction intervals. E: Dispersion (same as in panel A, B). Higher values mean greater dispersion of the forecast and imply ceteris paribus a worse score. F: Bias, i.e. general (relative) tendency to over- or underpredict. Values are between -1 (complete under-prediction) and 1 (complete over-prediction) and 0 ideally. G: Absolute error of the median forecast (lower is better). H. Standard deviation of WIS values. (TIF) [file pcbi.1010405.s015.tif]

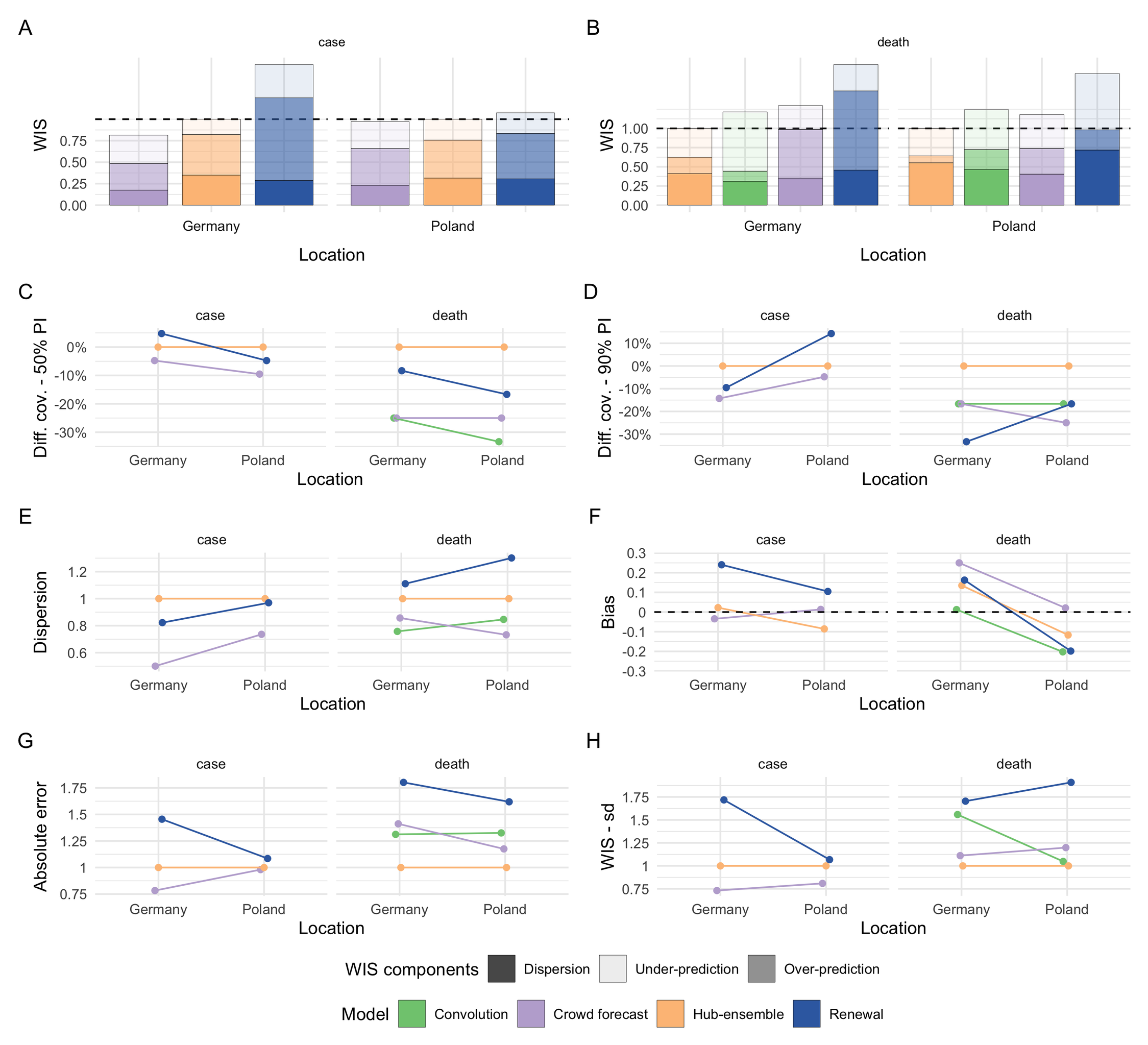

Supplement: S5 Fig — A, B: mean weighted interval score (WIS) across locations (lower values indicate better performance). C, D: Empirical coverage of the 50% and 90% prediction intervals. E: Dispersion. Higher values mean greater dispersion of the forecast and imply ceteris paribus a worse score. F: Bias, i.e. general (relative) tendency to over- orunderpredict. Values are between -1 (complete under-prediction) and 1 (complete over-prediction) and 0 ideally. G: Absolute error of the median forecast. H. Standard deviation of WIS values. (TIF) [file pcbi.1010405.s016.tif]

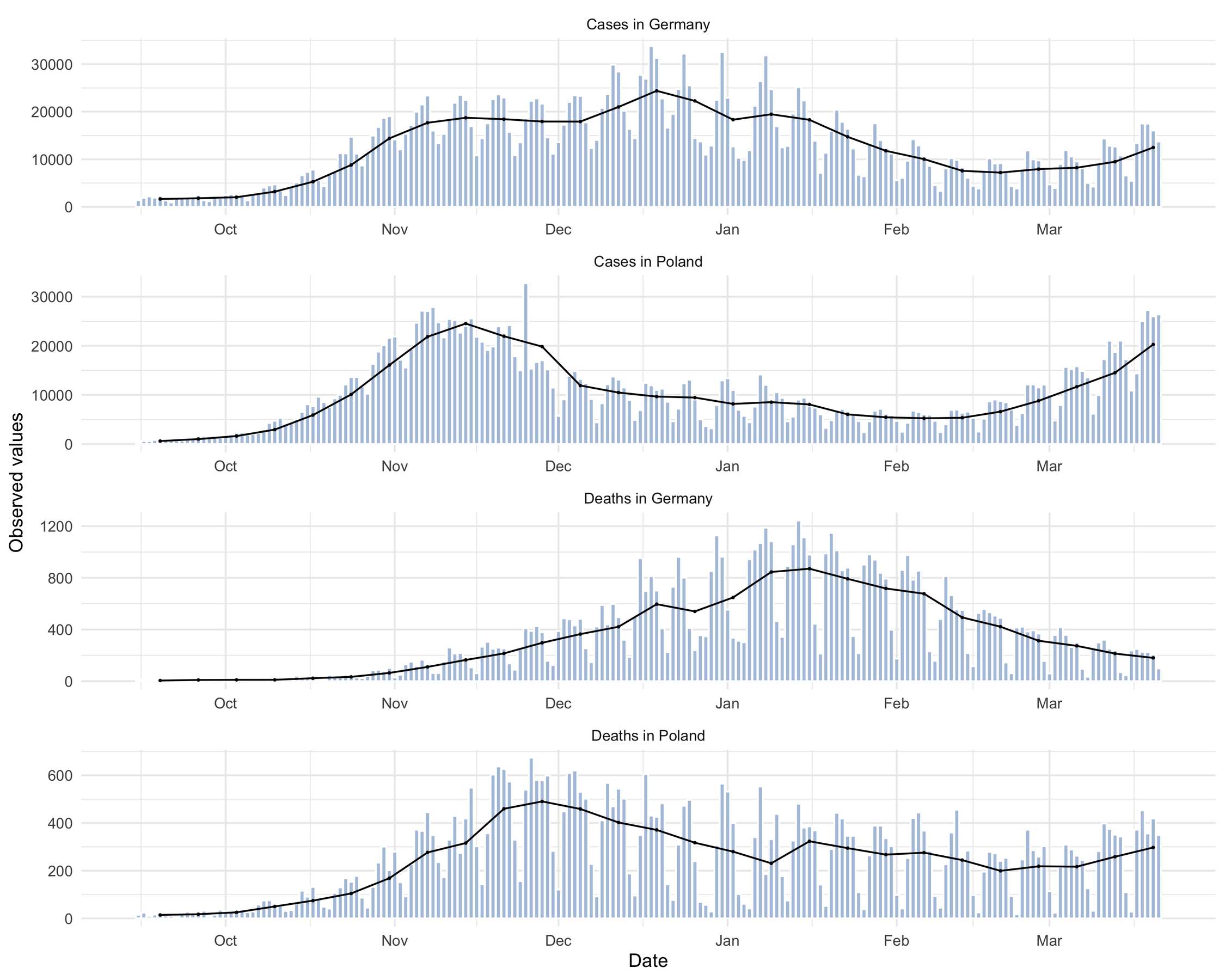

Supplement: S6 Fig — The black line represents weekly data divided by seven. Data were last accessed through the German and Polish Forecast Hub on August 21 2021. (TIF) [file pcbi.1010405.s017.tif]

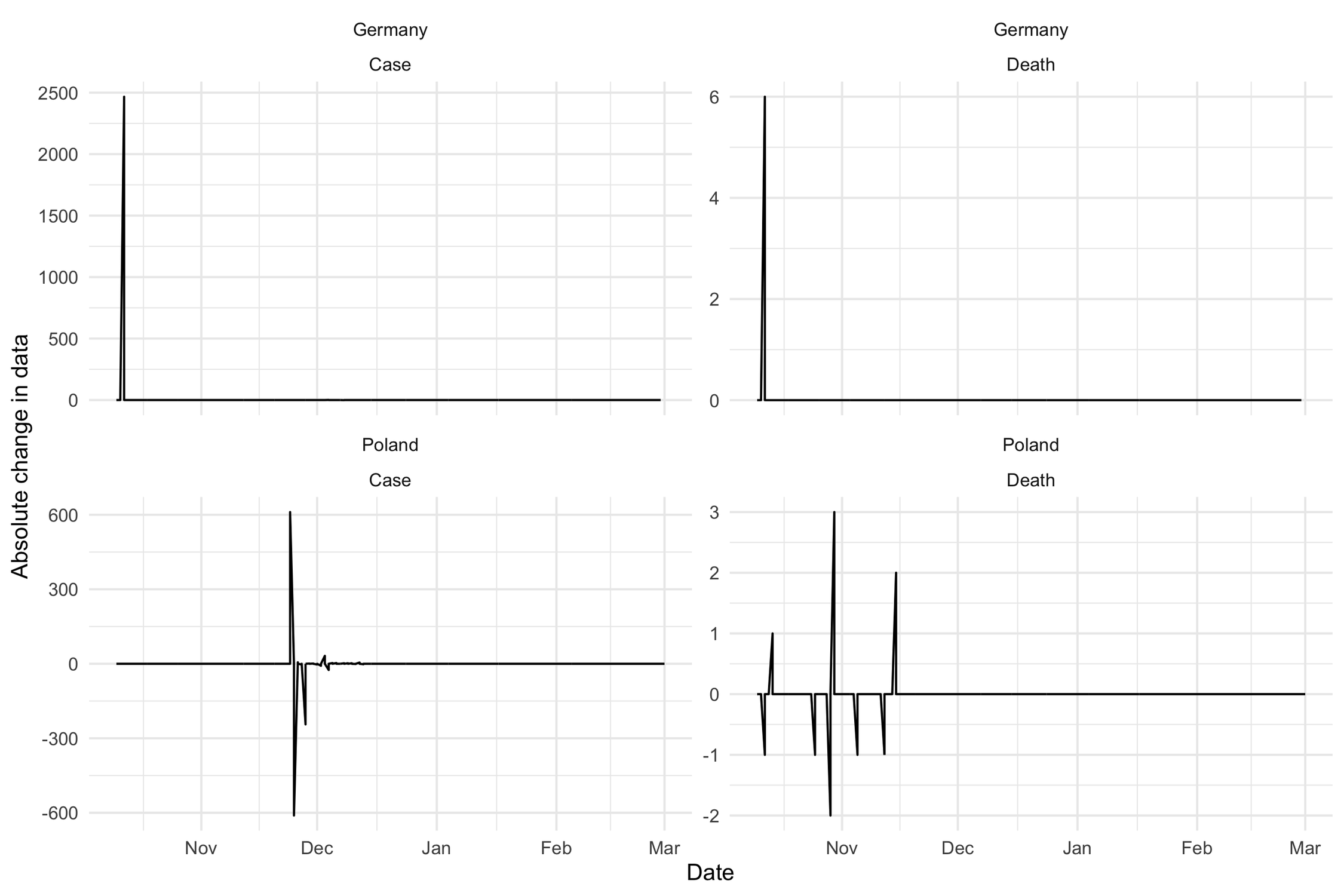

Supplement: S7 Fig — In Germany, there were zero cases and deaths reported on 2020–10-12, and only later 2467 cases and 6 deaths were added. Data were last accessed through the German and Polish Forecast Hub on May 10 2022. (TIF) [file pcbi.1010405.s018.tif]

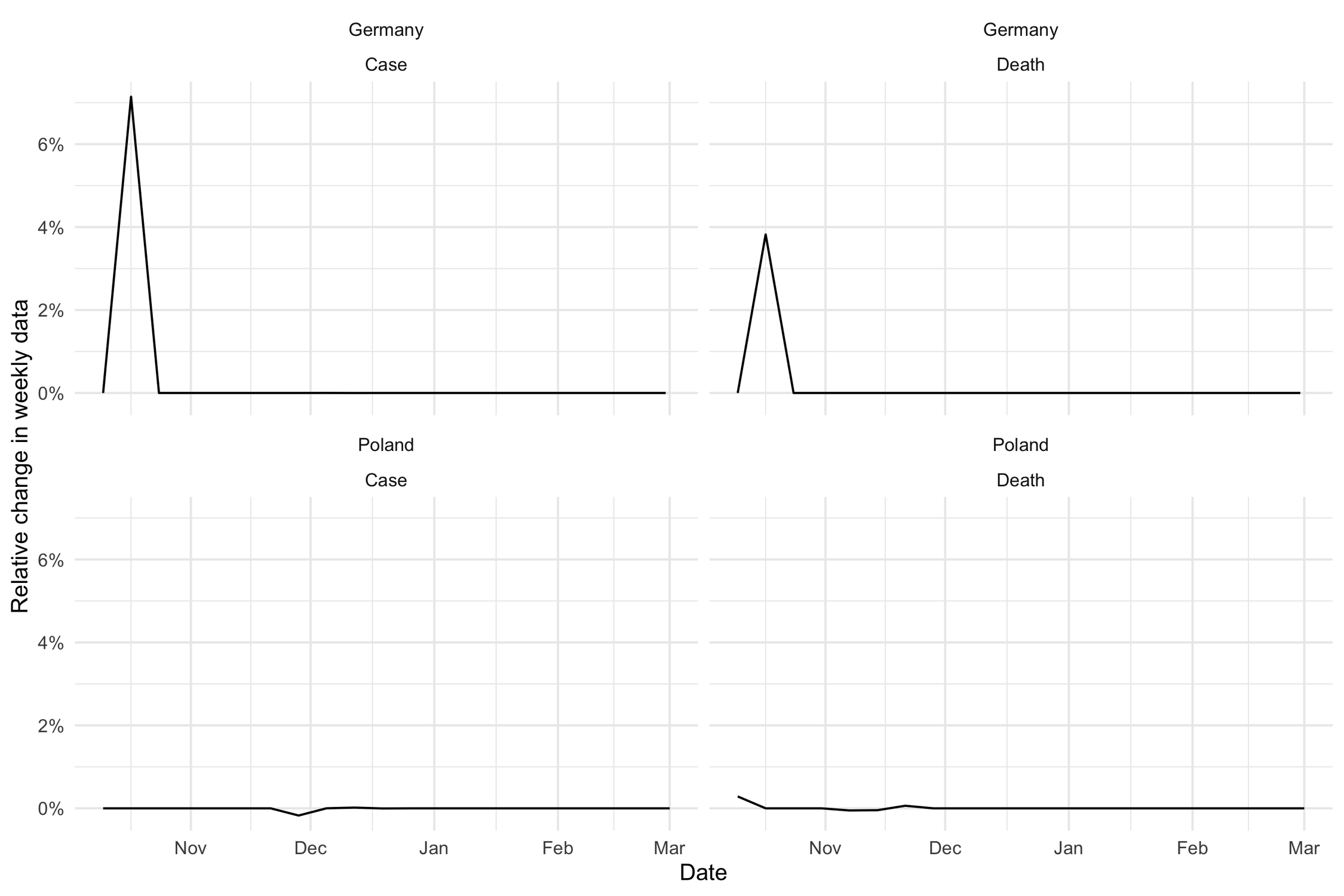

Supplement: S8 Fig — Apart from the data that was retrospectively added on 2020–10-12, data updates did not have a noticeable effect on weekly data (as shown in the forecasting application). Data were last accessed through the German and Polish Forecast Hub on May 10 2022. (TIF) [file pcbi.1010405.s019.tif]

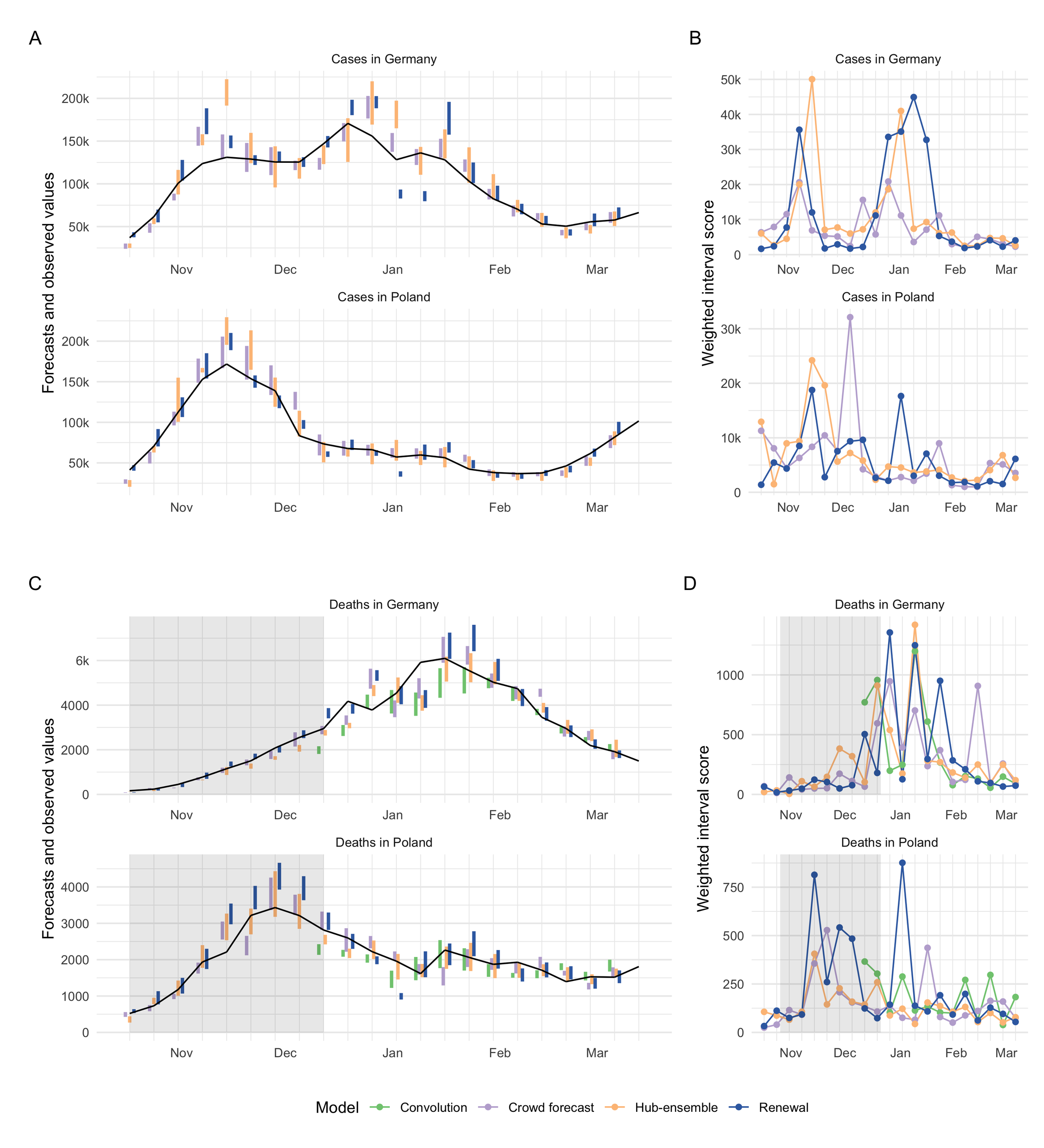

Supplement: S9 Fig — A, C: Visualisation of 50% prediction intervals of one week ahead forecasts against the reported values. Forecasts that were not scored (because there was no complete set of death forecasts available) are greyed out. B, D: Visualisation of corresponding WIS. (TIF) [file pcbi.1010405.s020.tif]

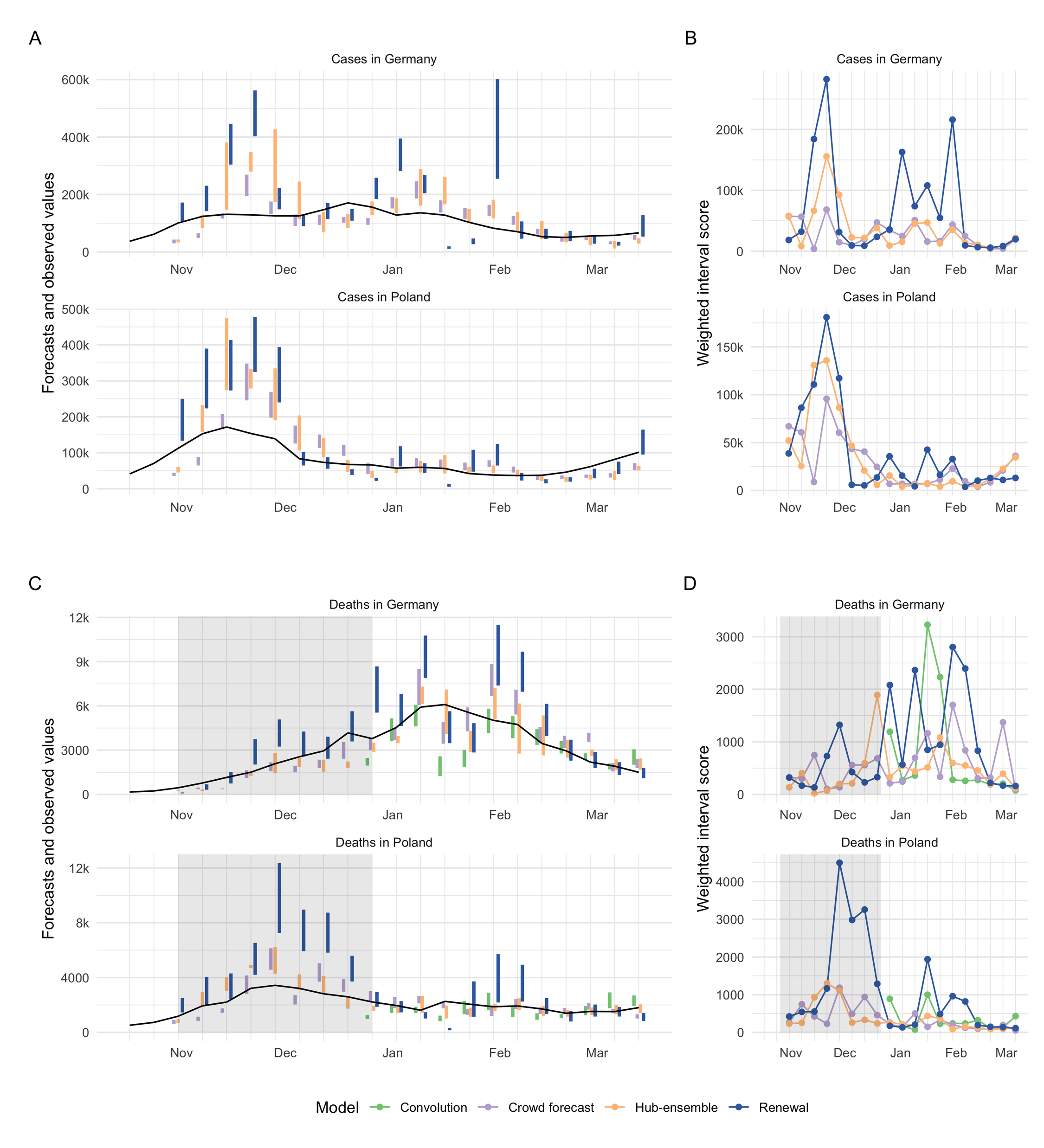

Supplement: S10 Fig — A, C: Visualisation of 50% prediction intervals of three week ahead forecasts against the reported values. Forecasts that were not scored (because there was no complete set of death forecasts available) are greyed out. B, D: Visualisation of corresponding WIS. (TIF) [file pcbi.1010405.s021.tif]

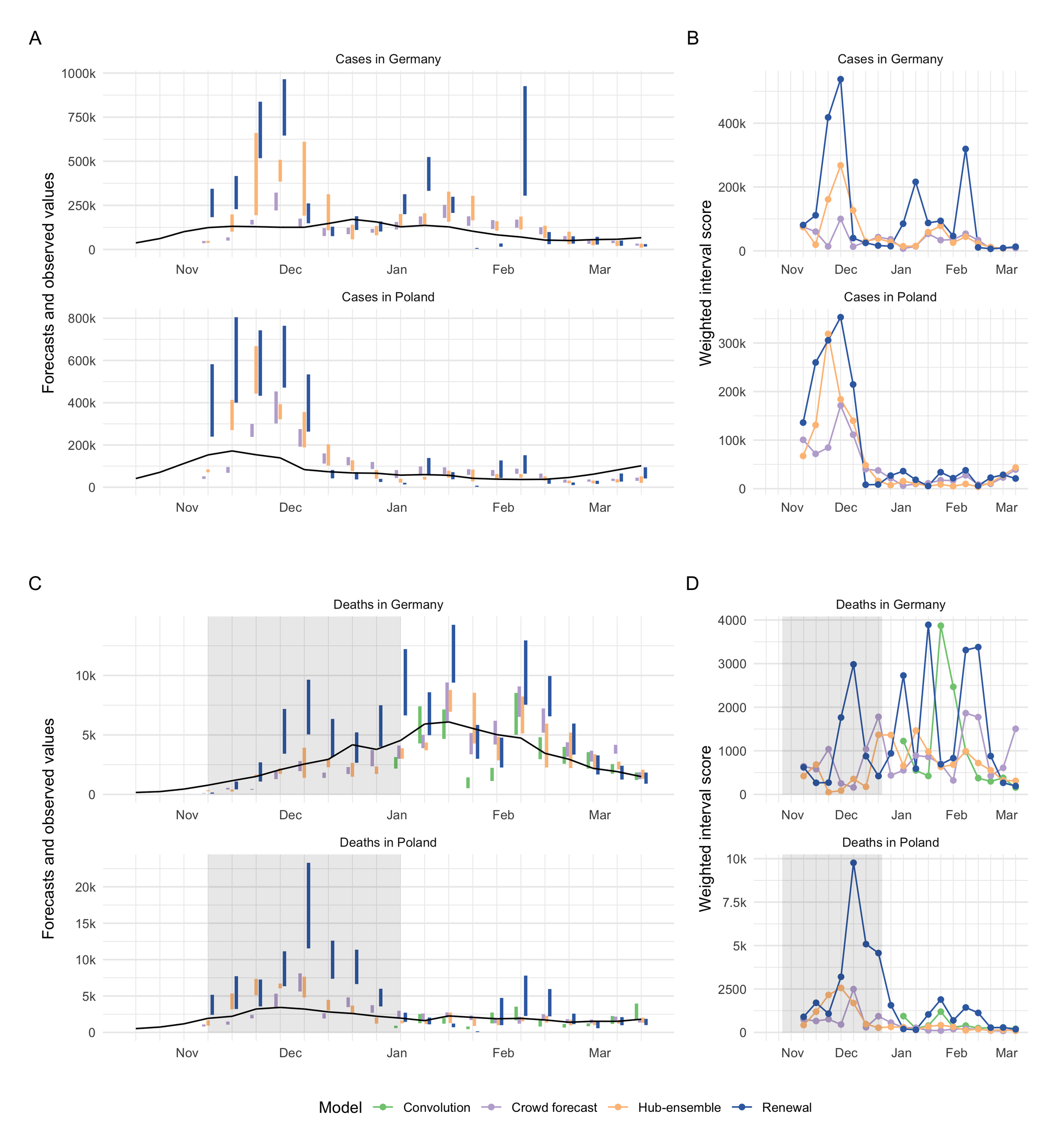

Supplement: S11 Fig — A, C: Visualisation of 50% prediction intervals of four week ahead forecasts against the reported values. Forecasts that were not scored (because there was no complete set of death forecasts available) are greyed out. B, D: Visualisation of corresponding WIS. (TIF) [file pcbi.1010405.s022.tif]

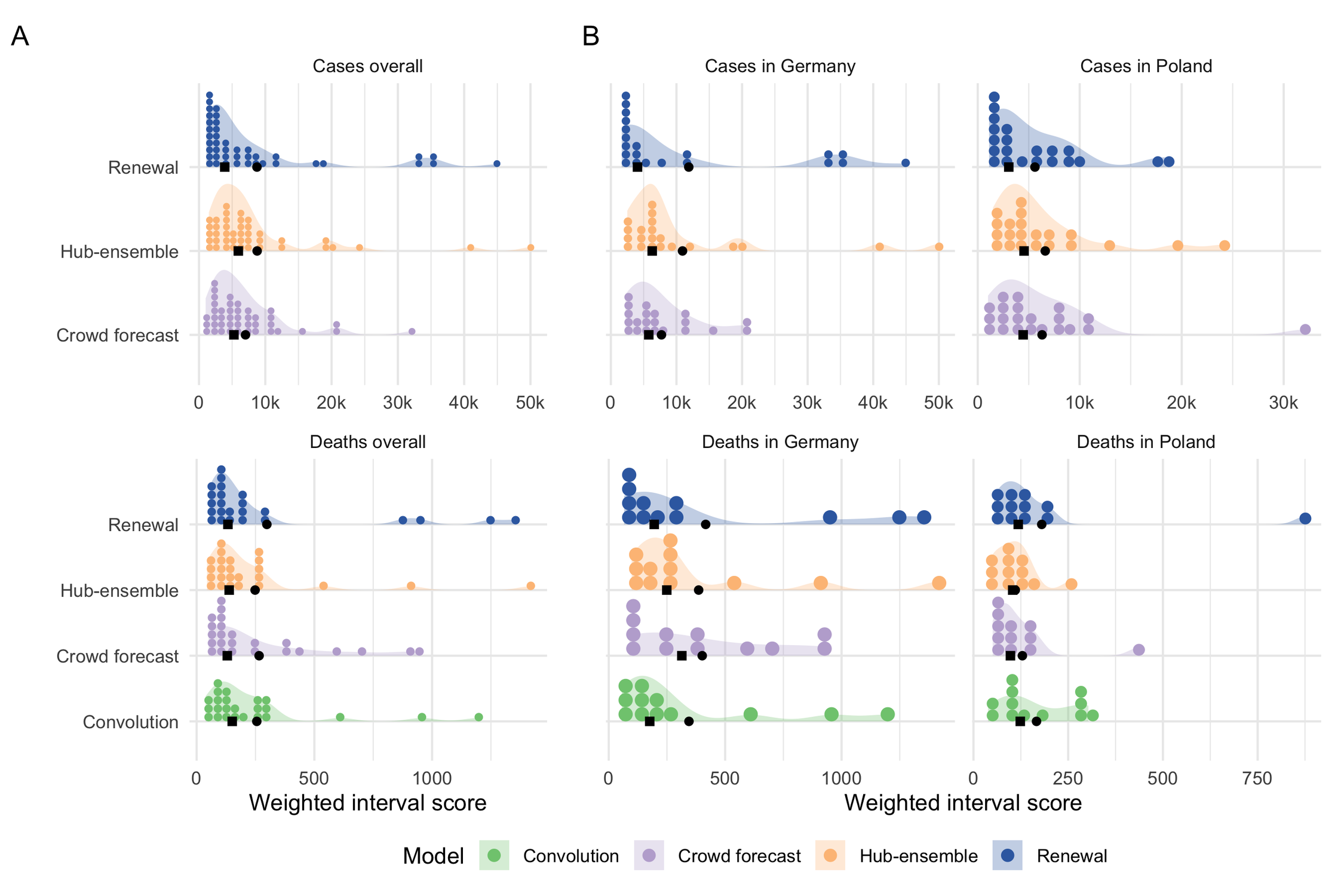

Supplement: S12 Fig — A: Distribution of weighted interval scores for one week ahead forecasts of the different models and forecast targets pooled across locations. B: Distribution of WIS separate by country. (TIF) [file pcbi.1010405.s023.tif]

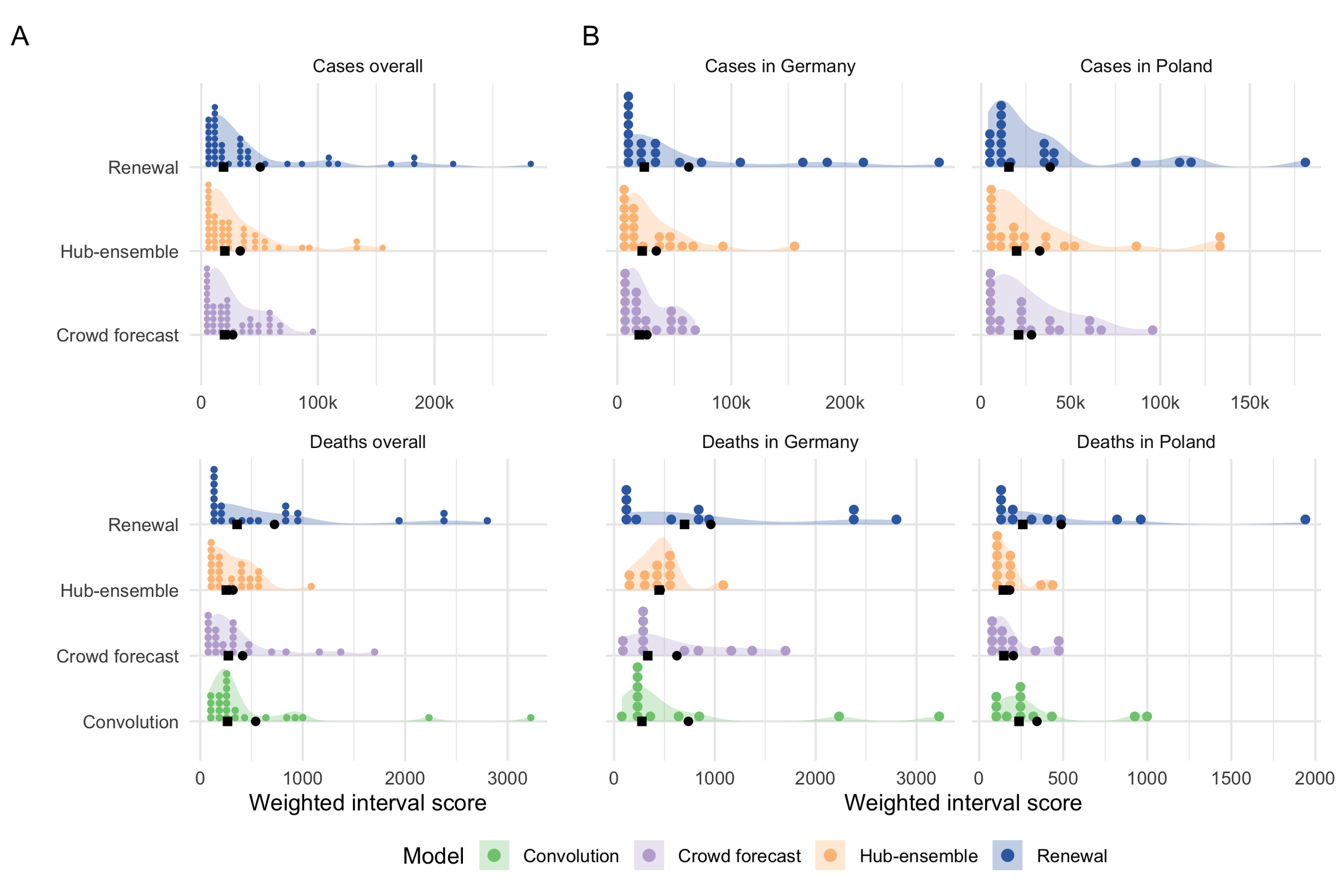

Supplement: S13 Fig — A: Distribution of weighted interval scores for three week ahead forecasts of the different models and forecast targets pooled across locations. B: Distribution of WIS separate by country. (TIF) [file pcbi.1010405.s024.tif]

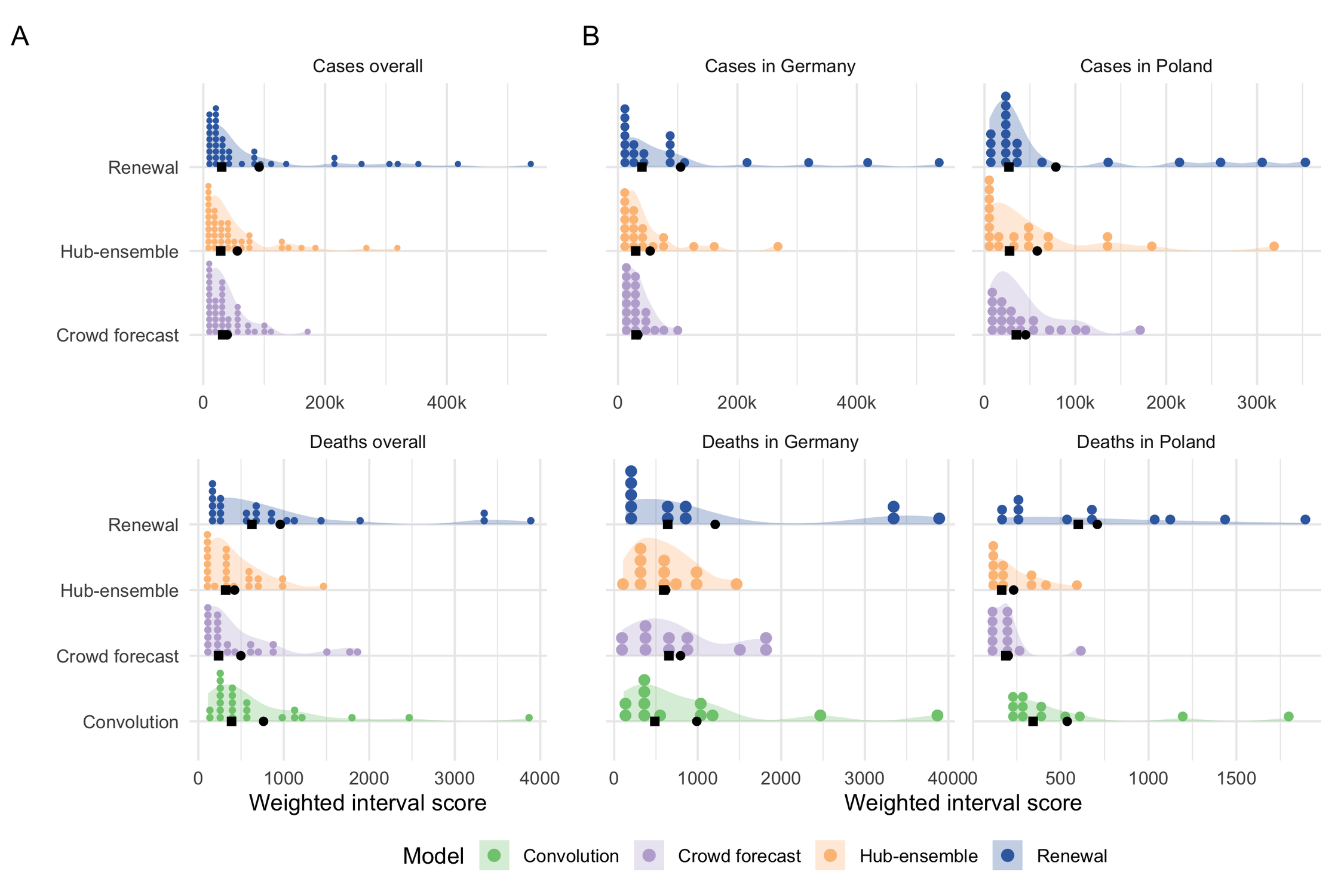

Supplement: S14 Fig — A: Distribution of weighted interval scores for four week ahead forecasts of the different models and forecast targets pooled across locations. B: Distribution of WIS separate by country. (TIF) [file pcbi.1010405.s025.tif]

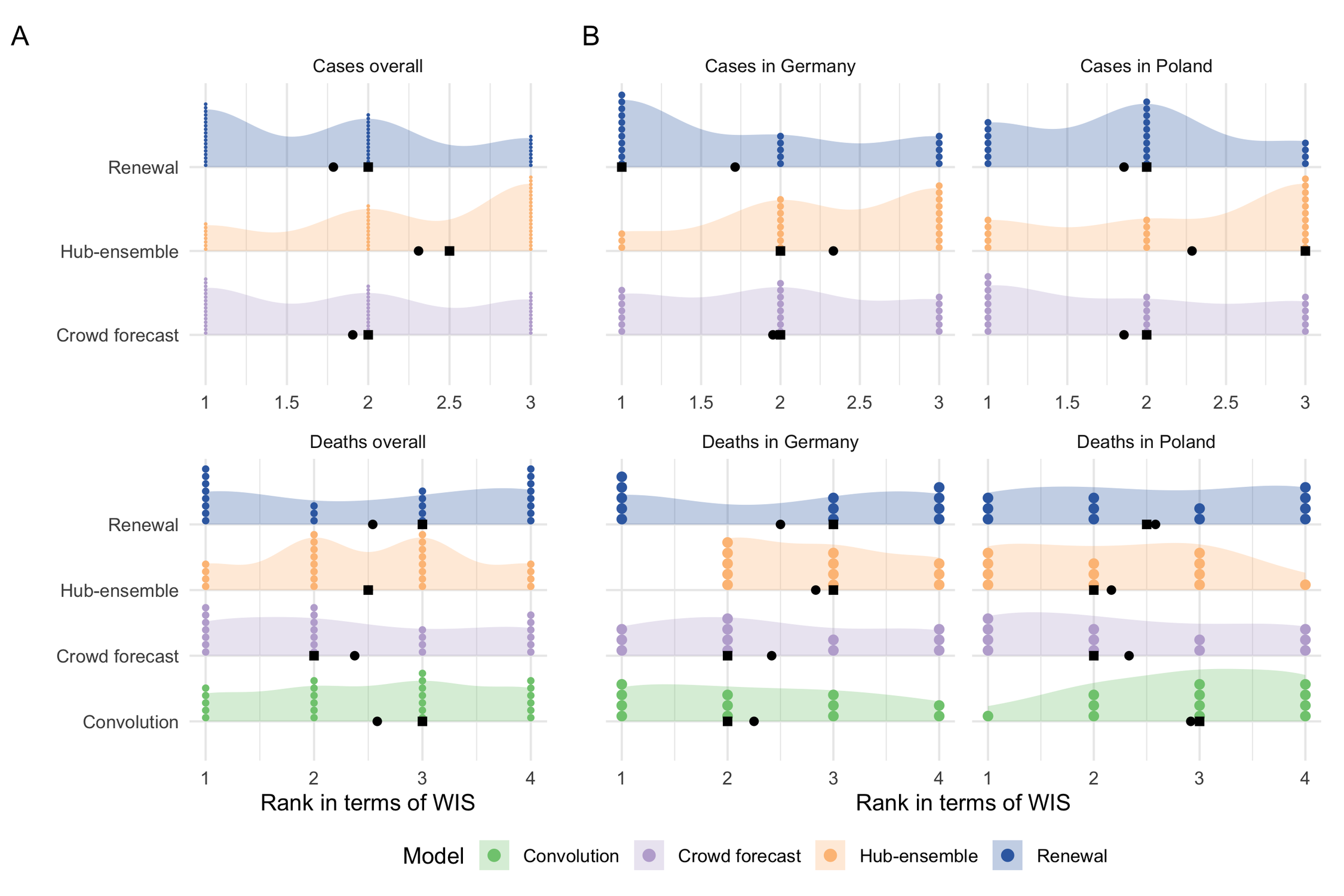

Supplement: S15 Fig — A: Distribution of the ranks (determined by the weighted interval score) for one week ahead forecasts of the different models and forecast targets, pooled across locations. B: Distribution of ranks separate by country. (TIF) [file pcbi.1010405.s026.tif]

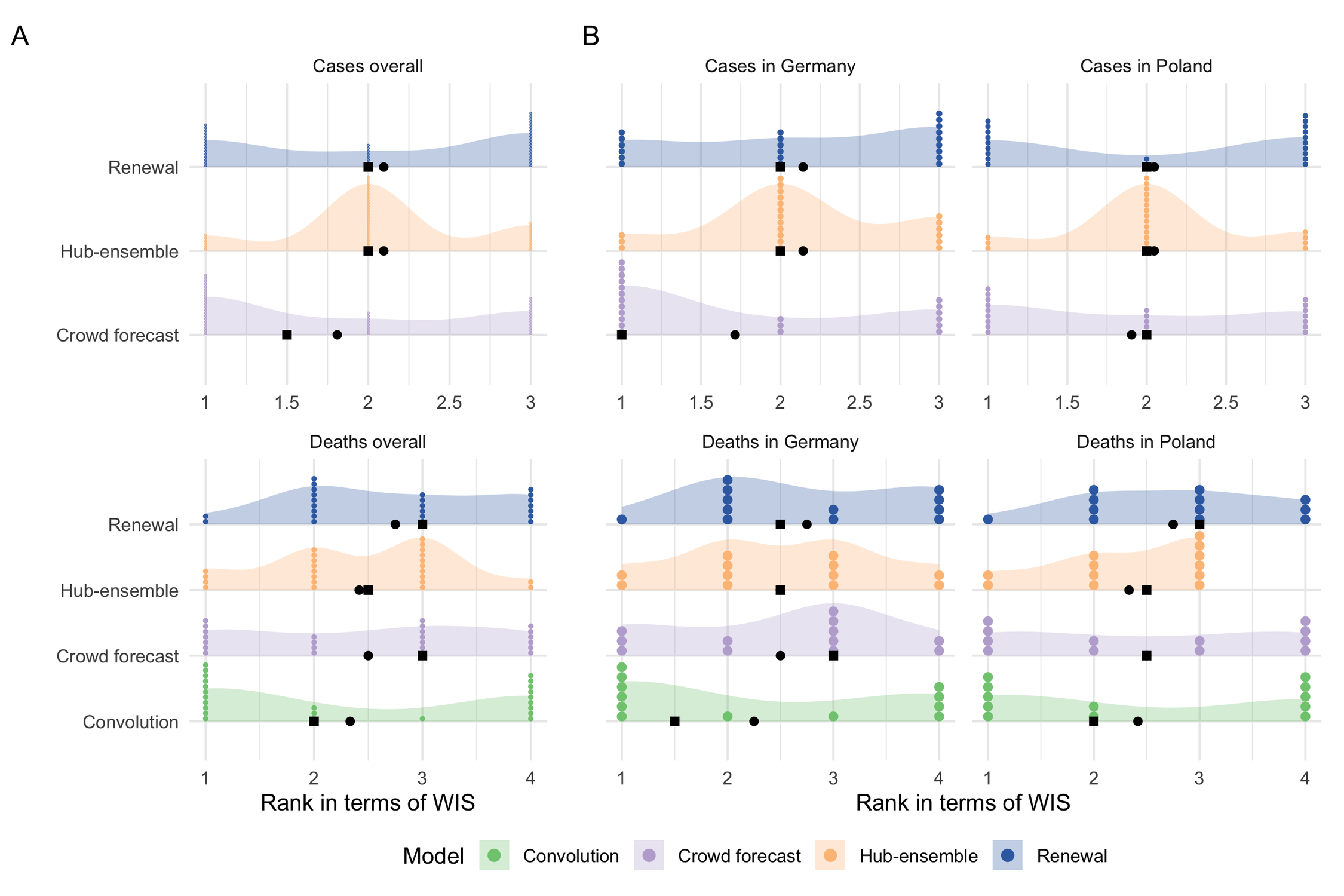

Supplement: S16 Fig — A: Distribution of the ranks (determined by the weighted interval score) for two week ahead forecasts of the different models and forecast targets, pooled across locations. B: Distribution of ranks separate by country. (TIF) [file pcbi.1010405.s027.tif]

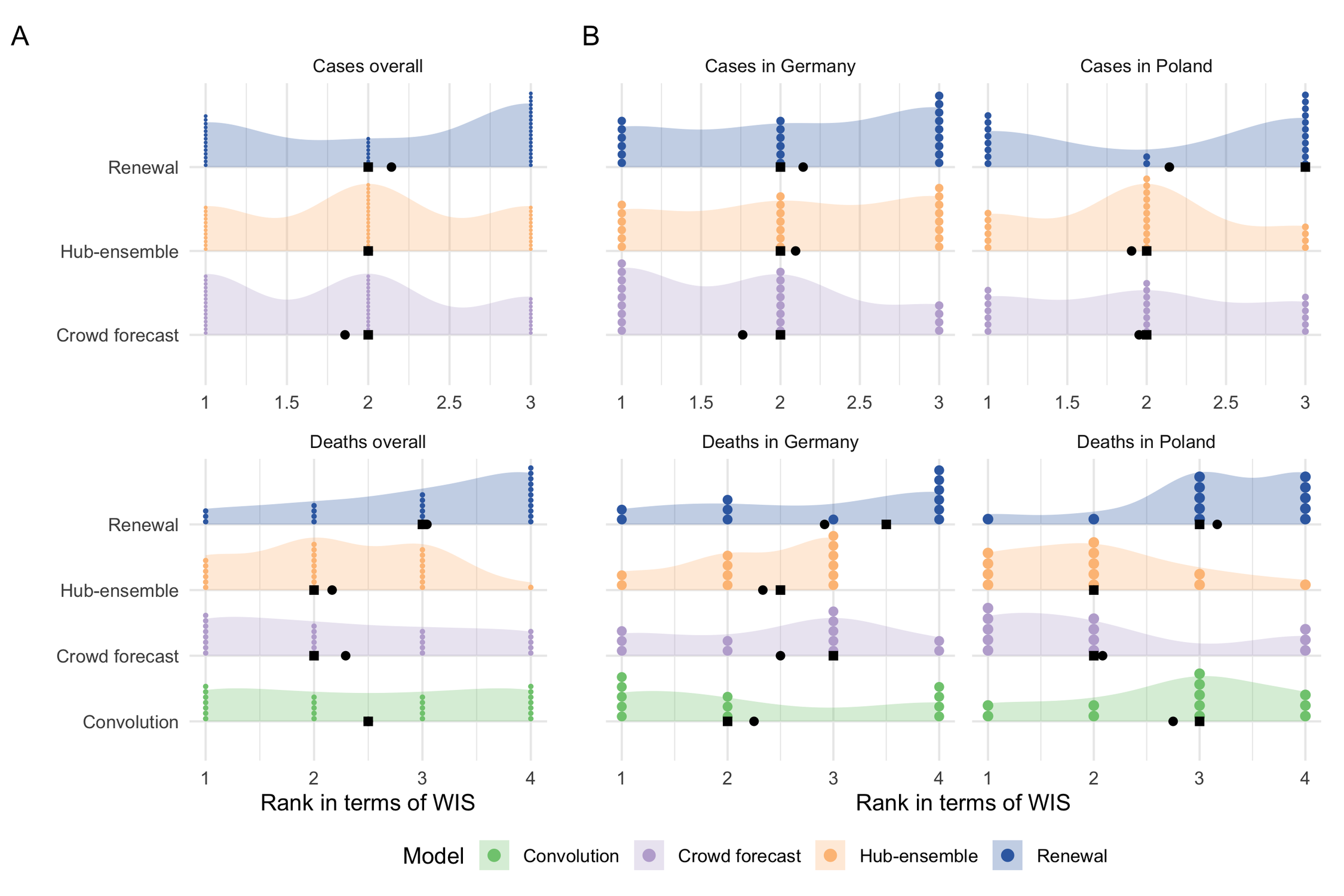

Supplement: S17 Fig — A: Distribution of the ranks (determined by the weighted interval score) for three week ahead forecasts of the different models and forecast targets, pooled across locations. B: Distribution of ranks separate by country. (TIF) [file pcbi.1010405.s028.tif]

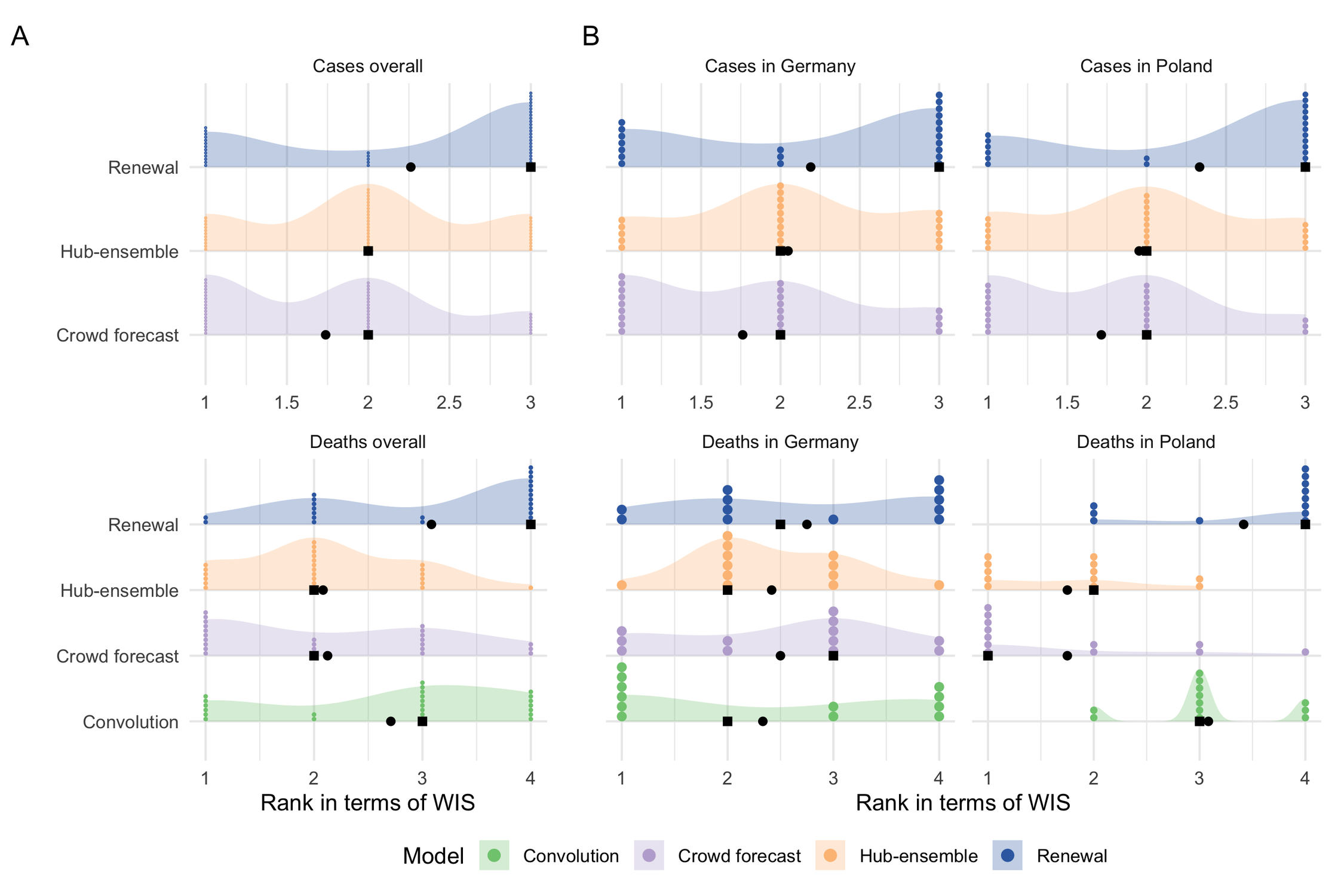

Supplement: S18 Fig — A: Distribution of the ranks (determined by the weighted interval score) for four week ahead forecasts of the different models and forecast targets, pooled across locations. B: Distribution of ranks separate by country. (TIF) [file pcbi.1010405.s029.tif]

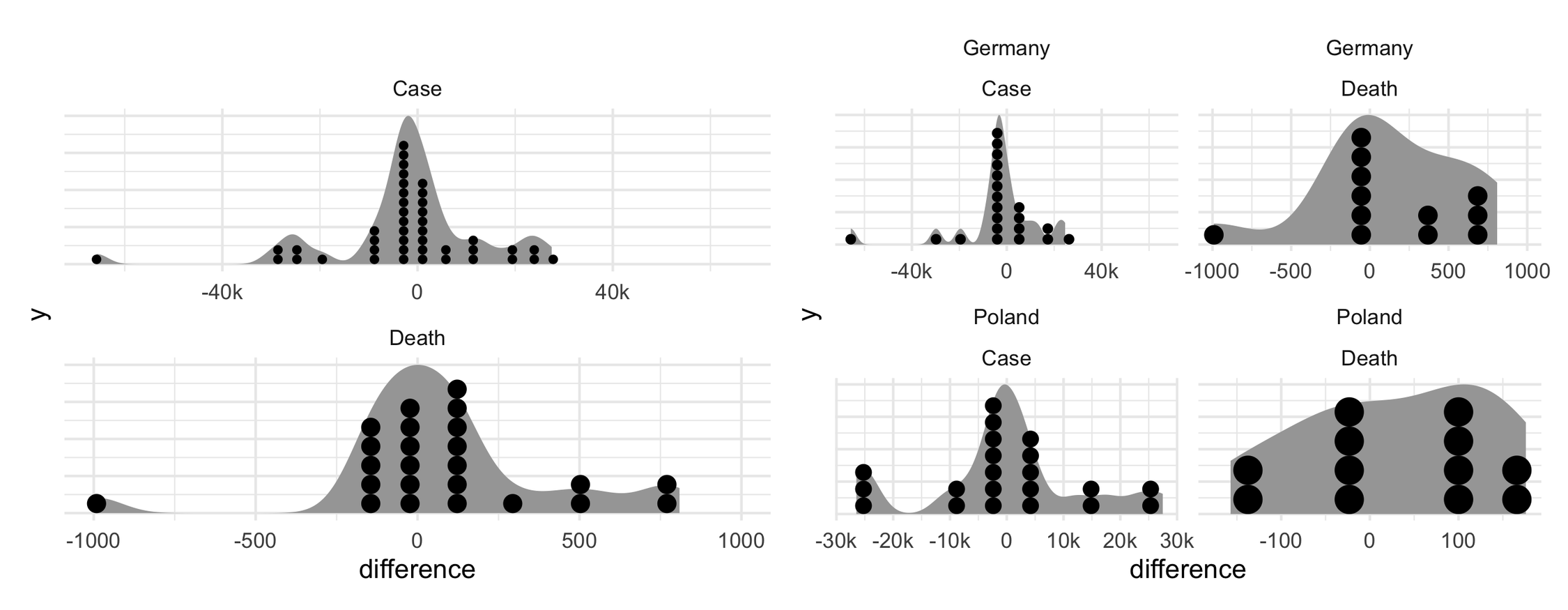

Supplement: S19 Fig — Values below zero mean better performance of the Crowd forecasts. (TIF) [file pcbi.1010405.s030.tif]

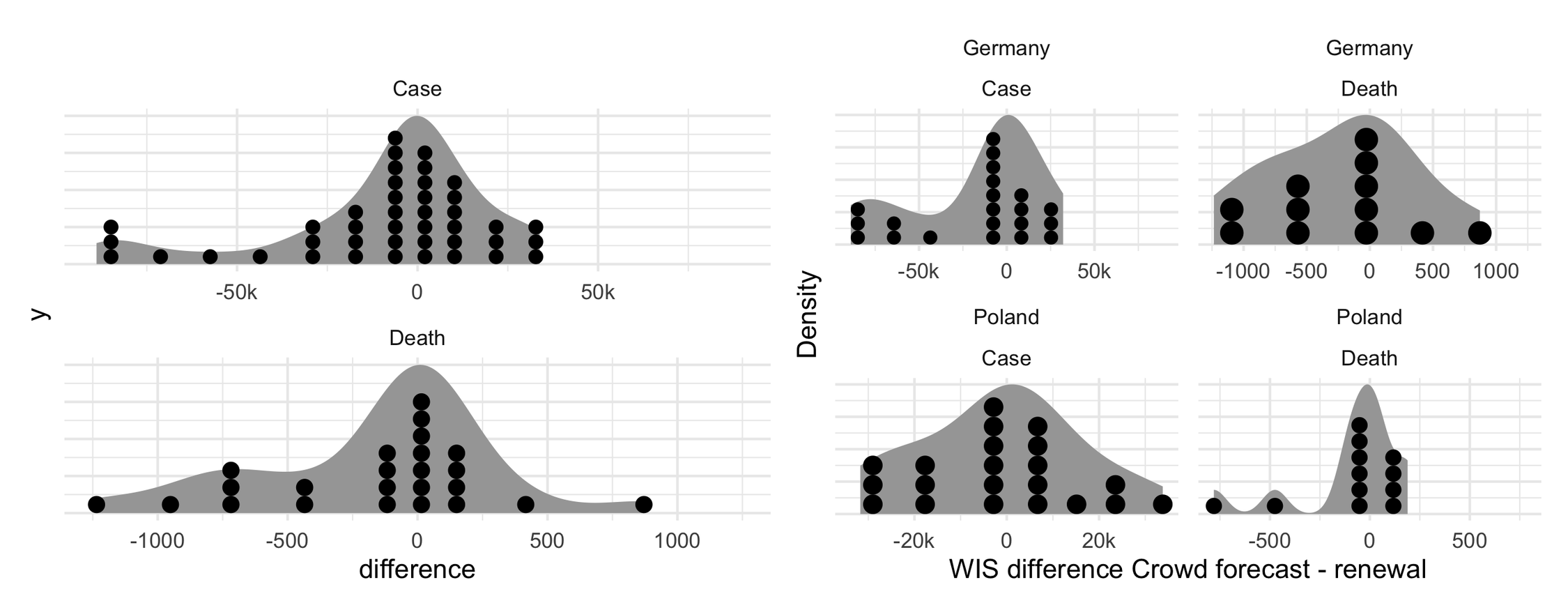

Supplement: S20 Fig — Values below zero mean better performance of the Crowd forecasts. (TIF) [file pcbi.1010405.s031.tif]

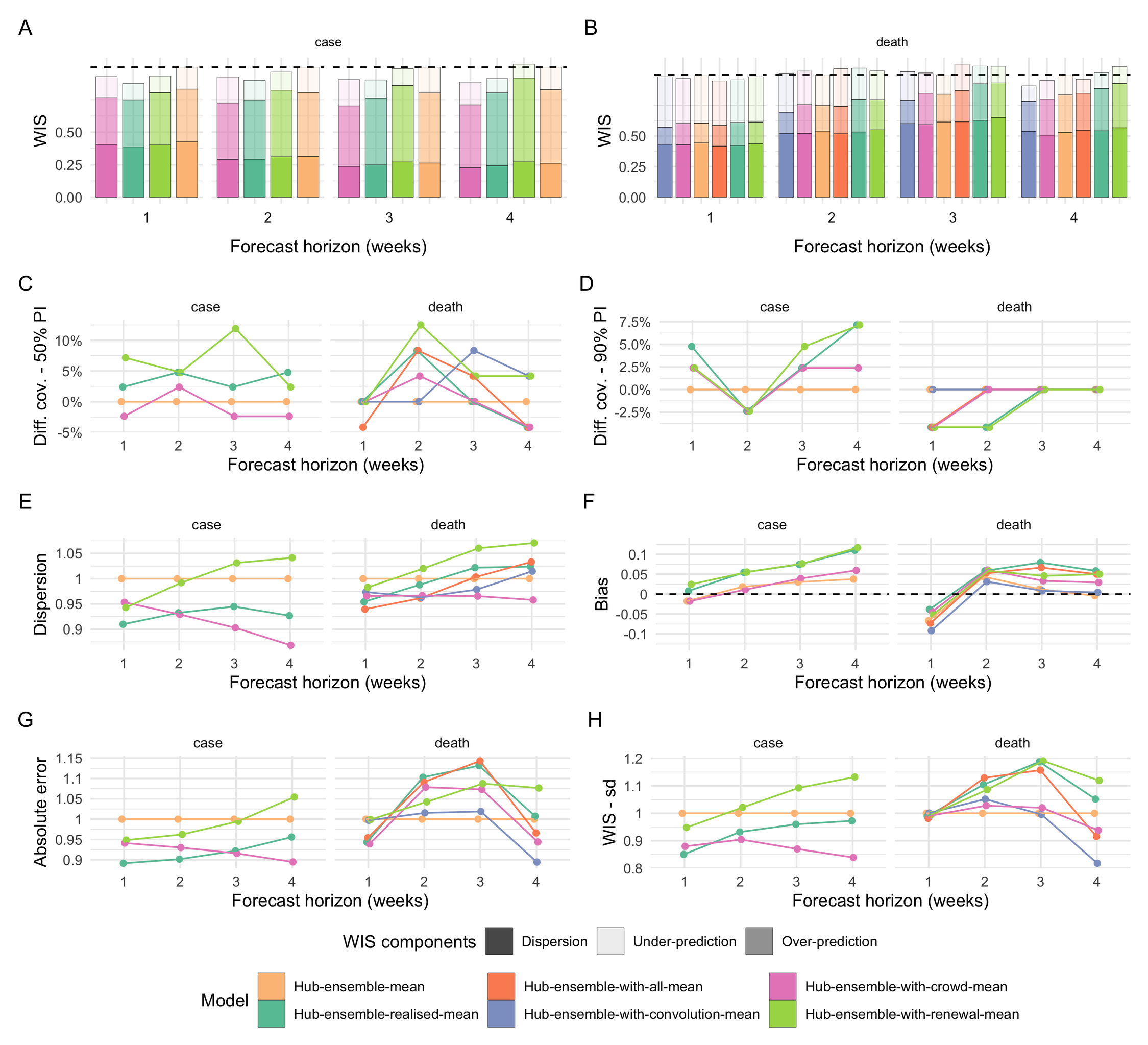

Supplement: S21 Fig — “Hub-ensemble” excludes all our models, Hub-ensemble-all includes all of our models, “Hub-ensemble-realised” is the actual hub-ensemble observed in reality, which includes the renewal model and the crowd forecasts, but ont the convolution model. Values (except for Bias) are computed as differences to the Hub ensemble which excludes our contributions. For Coverage, this is an absolute difference, for other metrics this is a percentage difference. A, B: mean weighted interval score (WIS) across horizons relative to the Hub ensemble (lower values indicate better performance). C, D: Empirical coverage of the 50% and 90% prediction intervals minus empirical coverage observed for the Hub ensemble. E: Dispersion relative to the dispersion of the Hub ensemble. Higher values mean greater dispersion of the forecast and imply ceteris paribus a worse score. F: Bias, i.e. general (relative) tendency to over- orunderpredict. Values are between -1 (complete under-prediction) and 1 (complete over-prediction) and 0 ideally. G: Absolute error of the median forecast relative to the Hub ensemble. H. Standard deviation of all WIS values for different horizons relative to the Hub ensemble. (TIF) [file pcbi.1010405.s032.tif]

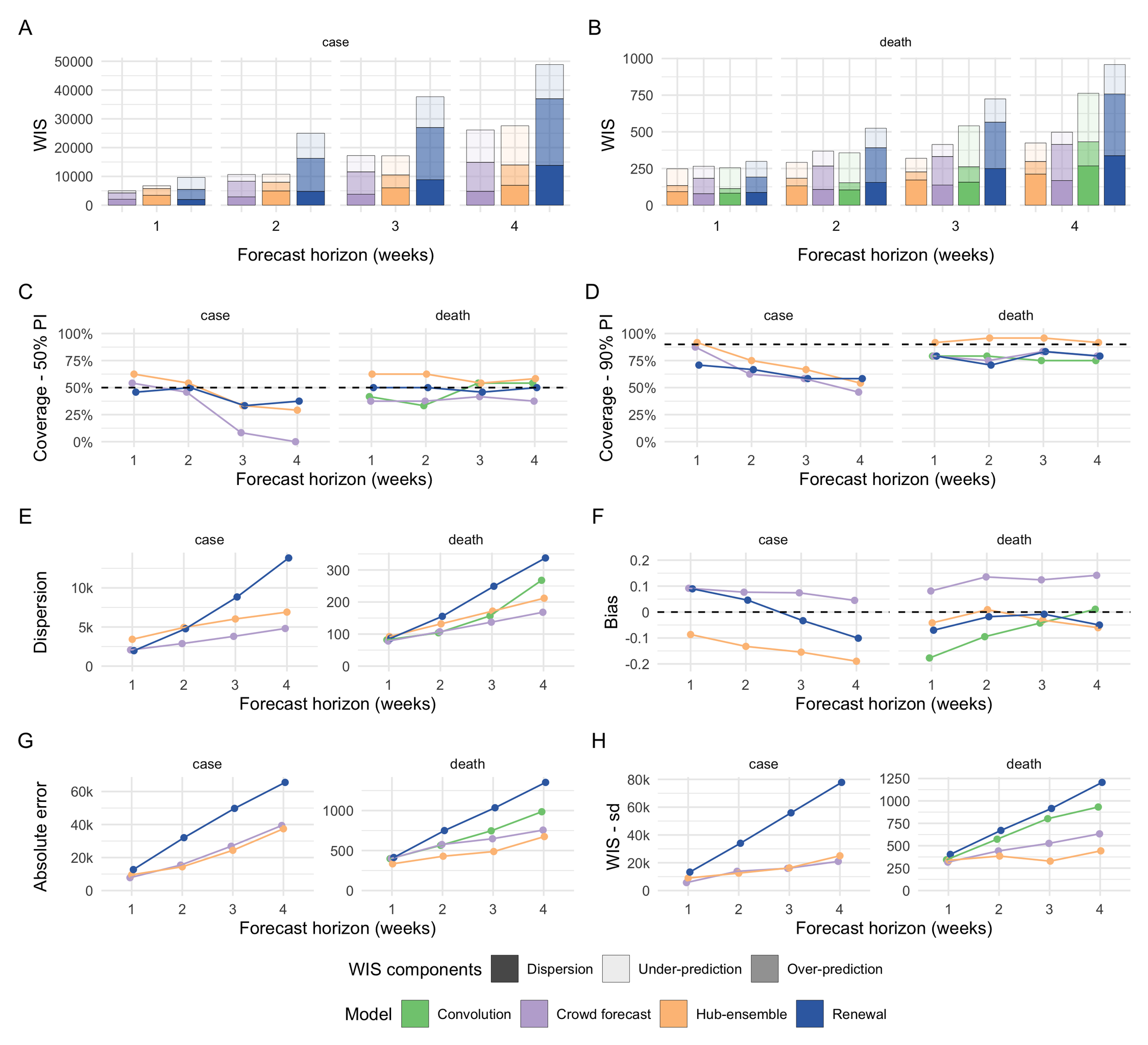

Supplement: S22 Fig — From December 14th 2020 on, all models were available. In the original analysis, cases and deaths were scored on different periods, as the convolution model was only added later. This sensitivity analysis shows performance of all models restricted to the period from December 14 2020 until March 1st 2021 where all models were available. A, B: mean weighted interval score (WIS, lower indicates better performance) across horizons. WIS is decomposed into its components dispersion, over-prediction and under-prediction. C: Empirical coverage of the 50% prediction intervals (50% coverage is perfect). D: Empirical coverage of the 90% prediction intervals. E: Dispersion (same as in panel A, B). Higher values mean greater dispersion of the forecast and imply ceteris paribus a worse score. F: Bias, i.e. general (relative) tendency to over- or underpredict. Values are between -1 (complete under-prediction) and 1 (complete over-prediction) and 0 ideally. G: Absolute error of the median forecast (lower is better). H. Standard deviation of all WIS values for different horizons (TIF) [file pcbi.1010405.s033.tif]

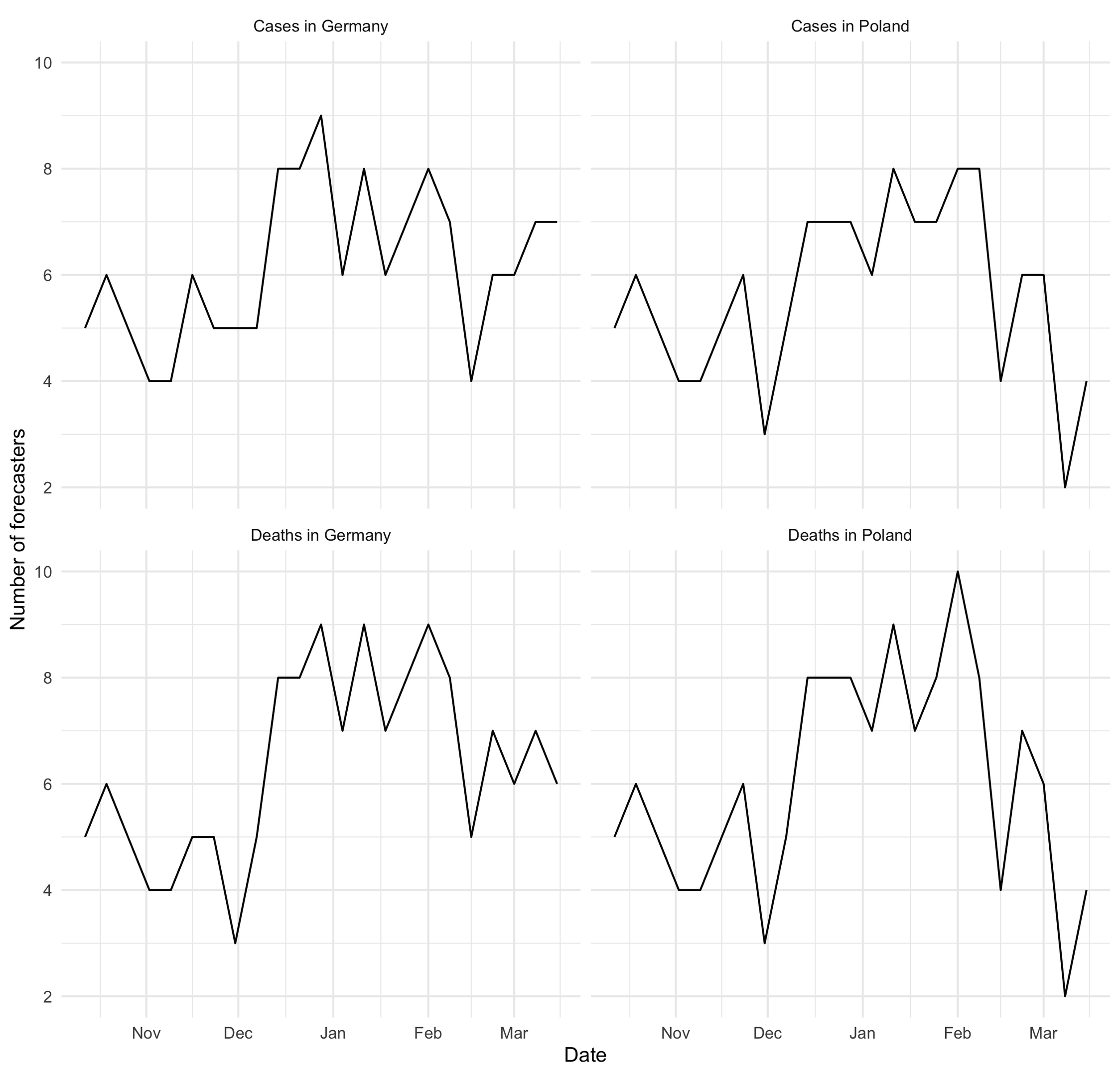

Supplement: S23 Fig — (TIF) [file pcbi.1010405.s034.tif]

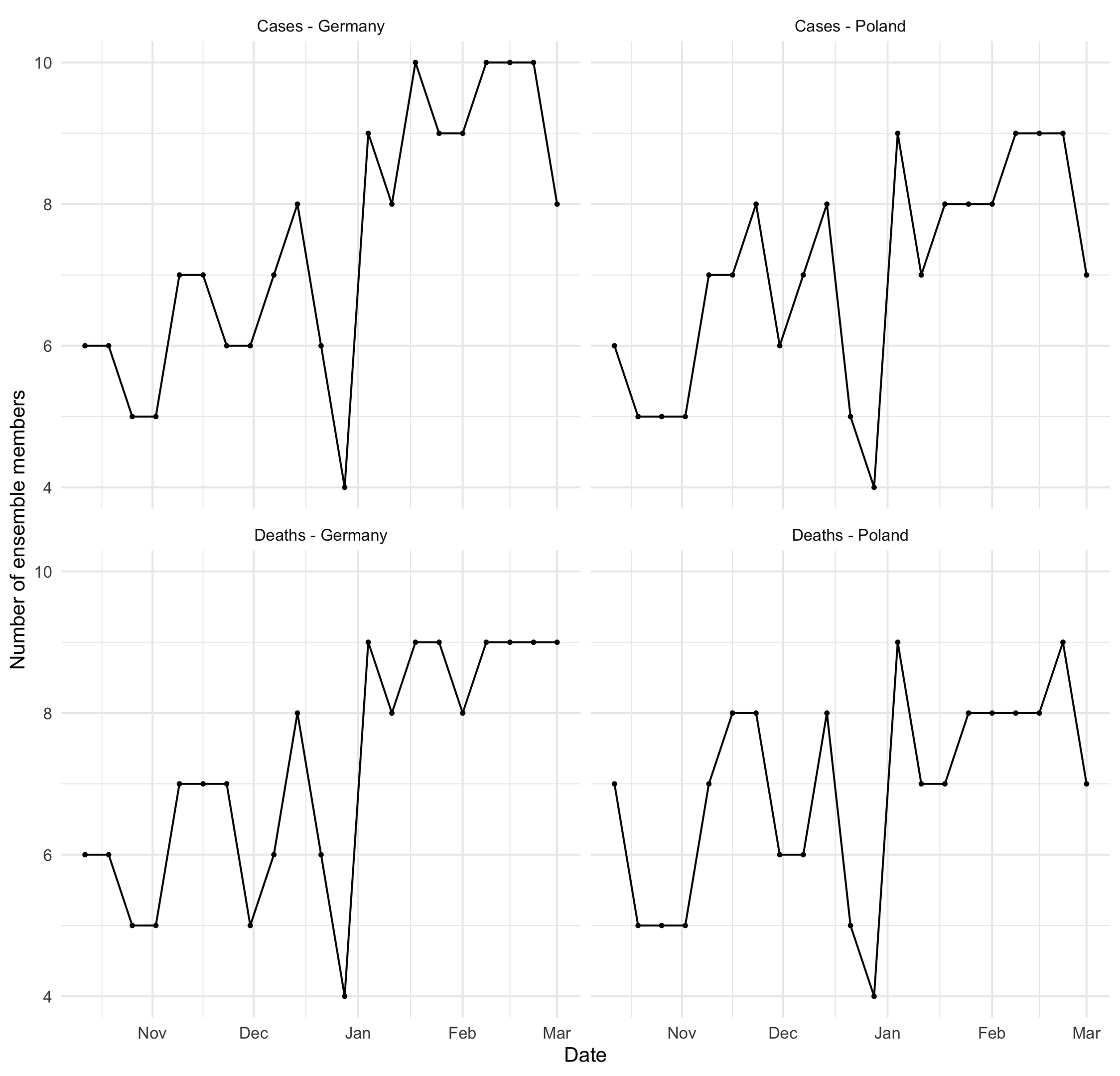

Supplement: S24 Fig — This includes our crowd forecasts and the renewal model. Note that the renewal model was not included in the ensemble on December 28th 2020. (TIF) [file pcbi.1010405.s035.tif]

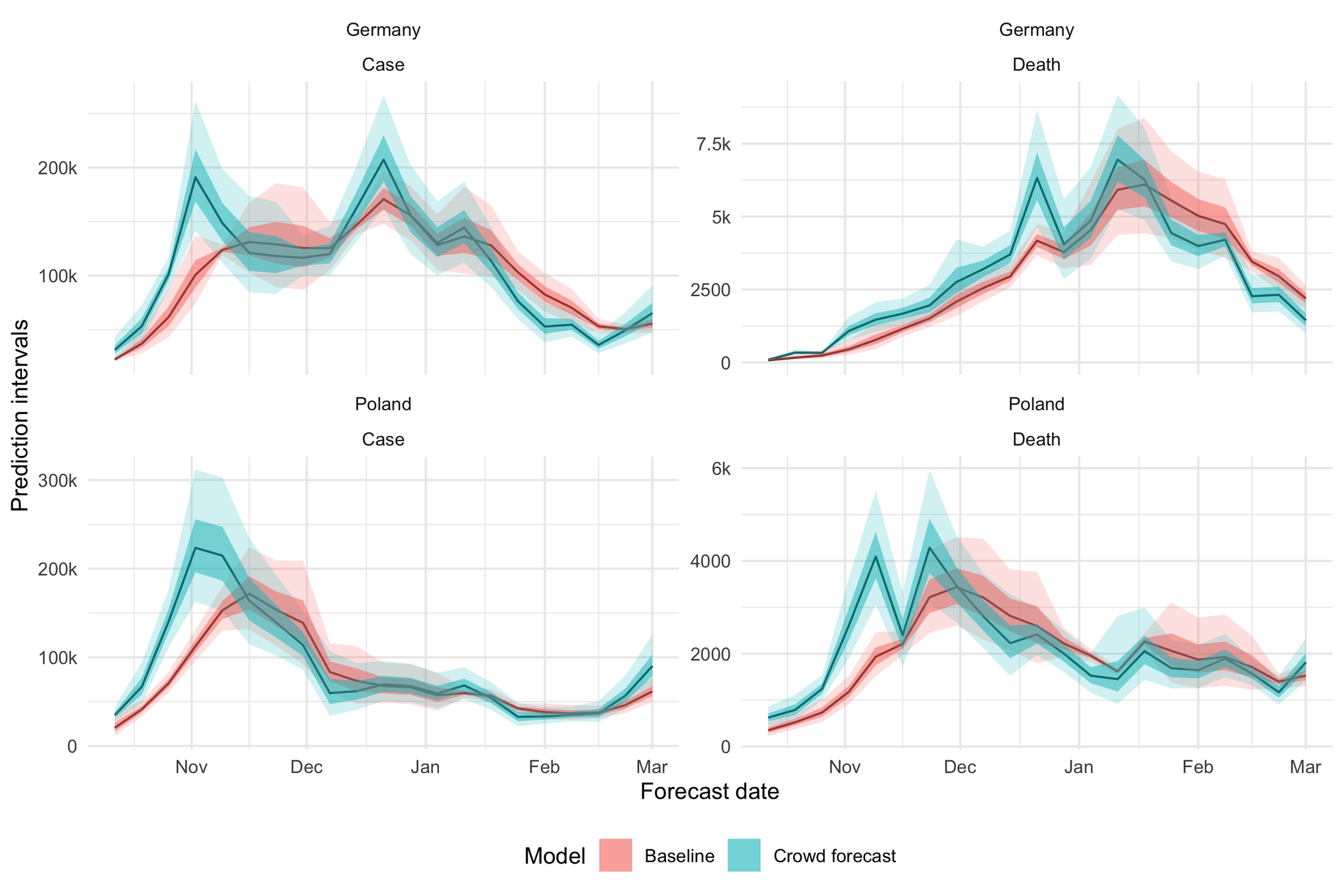

Supplement: S25 Fig — Shown are the median, as well as the 50% and 90% prediction intervals (in order of decreasing opacity). For any given point in time, the baseline shown in red is what forecasters saw when they opened the app (the baseline shown was constant across all forecast horizons). (TIF) [file pcbi.1010405.s036.tif]
