# Supplementary material for: Comparing human and model-based forecasts of COVID-19 in Germany and Poland
Source: PLoS Comput Biol. 2022 Sep 19;18(9):e1010405. doi: 10.1371/journal.pcbi.1010405 (PMC9534421; doi:10.1371/journal.pcbi.1010405)
Supplement: S1 Acknowledgements — (PDF) [file pcbi.1010405.s037.pdf]

We would like to acknowledge (in a randomised order) the members of Centre for the Mathematical Modelling of Infectious Diseases COVID-19 Working Group at the the London School of Hygiene & Tropical Medicine:

Oliver Brady, Katharine Sherratt, Kaja Abbas, Kerry LM Wong, Charlie Diamond, Katherine E. Atkins, Rein M G J Houben, Jiayao Lei, Rachel Lowe, David Simons, Sophie R Meakin, Nicholas G. Davies, Timothy W Russell, Kevin van Zandvoort, Quentin J Leclerc, Kathleen O'Reilly, Stéphane Hué, Alicia Rosello, Emilie Finch, C Julian Villabona-Arenas, Thibaut Jombart, W John Edmunds, Yalda Jafari, Jack Williams, Alicia Showering, Damien C Tully, Jon C Emery, Carl A B Pearson, David Hodgson, Frank G Sandmann, Petra Klepac, Adam J Kucharski, Graham Medley, Yang Liu, Simon R Procter, Emily S Nightingale, William Waites, Rosanna C Barnard, Joel Hellewell, Yung-Wai Desmond Chan, Fiona Yueqian Sun, Hamish P Gibbs, Rosalind M Eggo, Lloyd A C Chapman, Stefan Flasche, James W Rudge, Akira Endo, Naomi R Waterlow, Paul Mee, James D Munday, Ciara V McCarthy, Mihaly Koltai, Amy Gimma, Christopher I Jarvis, Megan Auzenbergs, Matthew Quaife, Fabienne Krauer, Samuel Clifford, Georgia R Gore-Langton, Arminder K Deol, Kiesha Prem, Gwenan M Knight, Rachael Pung, Anna M Foss.
