# Supplementary material for: Comparing human and model-based forecasts of COVID-19 in Germany and Poland
Source: PLoS Comput Biol. 2022 Sep 19;18(9):e1010405. doi: 10.1371/journal.pcbi.1010405 (PMC9534421; doi:10.1371/journal.pcbi.1010405)
Supplement: Attachment — Submitted filename: review.pdf [file pcbi.1010405.s038.pdf]

Comparing human and model-based forecasts of COVID-19 in Germany and Poland

PCOMPBIOL-D-22-00086

10 March 2022

This manuscript is well written and makes a useful contribution in evaluating judgmental crowd forecasts relative to models informed by epidemiological factors. The contribution provides all code and data, which I have used to reproduce your manuscript and results. My comments below predominantly consists of minor suggestions for improvements.

The expert epidemiologist forecasts used by policy makers are likely more complex than the model-based forecasts used as the comparison baseline. How does the performance of crowd-based forecasts (and ensembles that include them) compare with these expert forecasts?

Some analysis into how app defaults (especially for uncertainty) have influenced crowd forecasts would be useful to see. Are crowd forecasts generally 'overconfident' because of the default intervals? How much do crowd contributions differ from their default widths?

A better approach to computing crowd (and ensemble) forecast quantiles would be to use the quantiles from a mixture distribution of the forecasts, rather than averaging each contributing forecast quantile.

Some more information about which crowd forecasts were removed with manual visual inspection would strengthen the results. What were you looking in these forecasts to justify their removal? Would it be possible to remove infeasible crowd forecasts using thresholds rather than manual decision making?

A common approach for enhancing interpretability of comparing accuracy measures is to use a skill score. Rather than using $\frac{f(e)}{f(e_{hub})}$, using $1 - \frac{f(e)}{f(e_{hub})}$ is more widely used. With a skill score, equal performance occurs when the skill score is 0, and perfect forecasts give a score of 1.

There is a missing closing round bracket for 'Figure 3' in 5.2 paragraph 1.

Have you tried to explore the distribution of relative forecast performance, to see how likely it is for crowd forecasts to out perform model-based methods? It could be possible to bootstrap sample the weekly evaluations to get a sense of the significance of this result.

Were crowd forecasts purely based on human judgement, or could crowd forecast contributors use models or other mechanistic methods? The reporting of a leaderboard in the app could motivate the crowd forecast contributors to develop sophisticated models to improve performance, and I didn't notice any instructions advising contributors against doing this.
