# Supplementary material for: Comparing human and model-based forecasts of COVID-19 in Germany and Poland
Source: PLoS Comput Biol. 2022 Sep 19;18(9):e1010405. doi: 10.1371/journal.pcbi.1010405 (PMC9534421; doi:10.1371/journal.pcbi.1010405)
Supplement: Attachment — Submitted filename: Review comments German and Polish Forecasts.pdf [file pcbi.1010405.s039.pdf]

We thank the reviewers for their feedback and thoughtful comments which we feel have helped improve the manuscript substantially. Please see our responses to the individual comments as well as explanations on the changes we have made to the manuscript below.

Review #1

> The expert epidemiologist forecasts used by policy makers are likely more complex than the model-based forecasts used as the comparison baseline. How does the performance of crowd-based forecasts (and ensembles that include them) compare with these expert forecasts?

Our model-based forecasts (the renewal and the convolution) model were indeed slightly less complex than some of the models used to inform policy makers - but most importantly in the sense that they were not fine-tuned to the specific situations in Germany and Poland. That said, a version of the renewal model was used to estimate reproduction numbers and generate forecasts for the UK modelling committee SPI-M and continues to be used by the UK Health Security Agency (UKHSA) for real-time monitoring and thus ultimately supports policy making in the UK. Moreover, previous work on forecasting has found that complex mechanistic models usually do not outperform statistical models in predictive performance (e.g., Reich et al. https://www.pnas.org/doi/full/10.1073/pnas.1812594116 or Viboud et al. https://doi.org/10.1016/j.epidem.2017.08.002)

At the same time, at least some of the other models submitted to the German and Polish Forecast Hub were similar to the types of complex mechanistic models that we think you are alluding to. This is particularly the case for Poland, where models submitted by MOCOS and ICM were amongst those directly used to inform policy. We would argue that the comparison with the Hub-ensemble comes reasonably close to a comparison with models used by policy makers, but we agree that this topic is very interesting and important and warrants closer investigation. We added the following sentence to that effect to the Discussion (section 6): *"In light of the relatively small number of Hub ensemble models, performance of the Hub ensemble is also difficult to generalise. More research is needed to replicate these findings and investigate how crowd forecasts compare against the types of models and model ensembles policy makers use to inform their decisions."*

> Some analysis into how app defaults (especially for uncertainty) have influenced crowd forecasts would be useful to see. Are crowd forecasts generally 'overconfident' because of the default intervals? How much do crowd contributions differ from their default widths?

This is a very useful point and we have added a Figure to the SI (Figure S25) that compares the baseline forecast against the actual crowd forecast. The width of the default prediction intervals seems quite unrelated to the default baseline shown in the app and human forecasts tended to be noticeably sharper. We added the following sentence to that effect and a reference to that Figure in Section 4.2 and 5.2: "*A comparison of the crowd forecasts against the default baseline shown in the application is displayed in Figure S25 in the Appendix."*

Comparison of crowd forecasts and application baseline

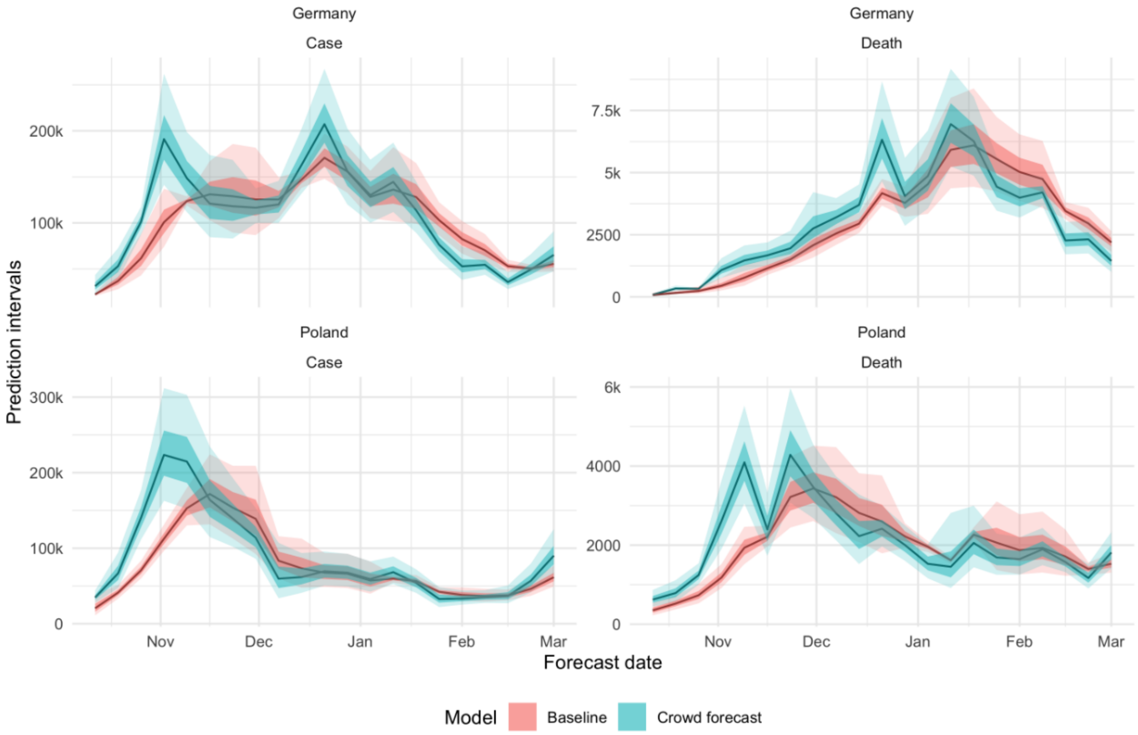

Fig S25. Crowd forecasts and baseline shown in the application for a two week horizon. Shown are the median, as well as the 50% and 90% prediction intervals (in order of decreasing opacity). For any given point in time, the baseline shown in red is what forecasters saw when they opened the app (the baseline shown was constant across all forecast horizons).

> A better approach to computing crowd (and ensemble) forecast quantiles would be to use the quantiles from a mixture distribution of the forecasts, rather than averaging each contributing forecast quantile.

We agree with you that it would be very interesting to analyse the performance of a mixture distribution ensemble, rather than just an ensemble based on quantile averages. However, we would argue that a priori it is an open question which of these two ensemble types performs better and to our best knowledge there is no clear consensus on it. There may also be additional issues where a mixture ensemble of well-calibrated models is not itself well calibrated. We decided for the quantile-average ensemble in our work for the following reasons:

- The quantile-average ensemble is the standard for the German and Polish Forecast Hub (as well as for the US Forecast Hub and now the European Forecast Hub).
- In order to create a mixture distribution ensemble we would have had to fit a distribution to the quantiles. The choice of the distribution is not obvious and would have introduced additional degrees of freedom

- Had we compared a mixture-distribution ensemble of crowd forecasts against the existing quantile-average ensemble from the Forecast hub, then any difference in performance between humans and models would have been confounded by the choice of the ensembling method

  Some more information about which crowd forecasts were removed with manual visual inspection would strengthen the results. What were you looking in these forecasts to justify their removal? Would it be possible to remove infeasible crowd forecasts using thresholds rather than manual decision making?

We did not remove many forecasts, but unfortunately we have not stored complete accounts of predictions that were removed. However, we only deleted predictions that were clearly the result of a user or software error, for example a forecast that was zero everywhere. Automatic quality checks of course have the advantage that criteria can be easily documented. The only automatic check we installed was whether a forecaster had submitted a forecast for at least two targets for a given forecast date. We did this to avoid situations in which someone would just visit the app to try it out and submit a random prediction. We added the following to the description in Section 4.2 to make this clearer. "*To avoid issues with users trying out the app and submitting a random forecast, we required that a forecaster needed to make a forecast for at least two targets for a given forecast in order to be included in the crowd forecast ensemble. On a few occasions we deleted forecasts that were clearly the result of a user or software error (such as for example forecasts that were zero everywhere).*"

A common approach for enhancing interpretability of comparing accuracy measures is to use a skill score. Rather than using $f(e) / f(e\_hub)$, using $1 – f(e) / f(e\_hub)$ is more widely used. With a skill score, equal performance occurs when the skill score is 0, and perfect forecasts give a score of 1.

We agree that in many fields the skill formulation you suggested is indeed more common and had thought about using it, but have decided for the current version for two main reasons:

- For all other measures that we report in the paper and the Figures like the WIS and its components, larger values imply worse performance. We thought it would be more confusing to mix these measures with a positively oriented skill score where greater values mean better performance.
- Several projects related to Forecast Hubs e.g. Cramer et al. (2022, https://www.pnas.org/doi/10.1073/pnas.2113561119) or Ray et al. (2022, https://arxiv.org/pdf/2201.12387.pdf), as well as other authors (e.g. Meakin et al. 2022, https://bmcmedicine.biomedcentral.com/articles/10.1186/s12916-022-02271-x) have used the present notation and thus using the same notation will make it easier for readers to be able to easily compare results.

There is a missing closing round bracket for 'Figure 3' in 5.2 paragraph 1.

We fixed it. Thank you.

Have you tried to explore the distribution of relative forecast performance, to see how likely it is for crowd forecasts to out perform model-based methods? It could be possible to bootstrap sample the weekly evaluations to get a sense of the significance of this result.

We agree this is interesting and added two new plots (Figures S19 and S20) to the SI to visualise the distribution of differences in WIS for the crowd forecasts against the Hub ensemble and the renewal model.
We also conducted a Wilcoxon-Mann-Whitney test to determine significance. Across all horizons, we found the following p-values:

Crowd forecast vs. Hub-ensemble: 0.09
Crowd forecast vs. Renewal: 0.0002
Crowd forecast vs. Convolution: 0.4 (fewer observations)

However, this is likely flawed, as forecasts made on a single day are correlated a) across horizons and b) across locations (e.g. human forecasters may have a good day or a bad day where they don't put a lot of effort into their forecasts), making it difficult to trust results.
When stratified across horizons, we only found p-values <0.05 for horizons 3 and 4 for the comparison between the Crowd forecast and the Renewal nodel.
We have not included these results in the paper, because we would like to avoid the impression that conclusions are immediately generalisable to other settings and all the other known issues associated with using p-values.

Were crowd forecasts purely based on human judgement, or could crowd forecast contributors use models or other mechanistic methods? The reporting of a leaderboard in the app could motivate the crowd forecast contributors to develop sophisticated models to improve performance, and I didn't notice any instructions advising contributors against doing this.

Forecasters could look at model-based predictions and at least one author of the study regularly looked at forecasts from the Renewal model before making their own prediction. Another forecaster often displayed the data on a log-scale in the app and held a ruler against the screen to "model" exponential growth. We are not aware of anyone using anything more sophisticated than that, but in principle it would have been permitted.
While we did not have an official leaderboard in the app, we did publish weekly reports showing past forecasts and scores on our website, epiforecasts.io. We added the following sentence to Section 4.2. to clarify this: "*From November 26 2020 on we displayed weekly small reports with a visualisation of past forecasts and scores on our website, epiforecasts.io.*"

Review #2.

1. It would be extremely interesting to see whether a larger study would support some of the observations reported here, such as model-based forecasts performing better for deaths than for cases (especially at longer forecast horizons) when compared to crowd forecasts.

We thank you for your kind words and we agree that these are very interesting questions and that a replication of the results observed here would be helpful. We plan to publish a paper about a follow-up study done in the UK which included a greater number of participants (but did not look at longer forecast horizons), which will hopefully allow us to revisit the topic.

2. Did the most recent case/death counts for a given forecasting week change in the data as reported in subsequent week(s)?
For example, in Figure 2C (Deaths in Poland) the crowd forecast around the start of December undershoots the reported values for a single week, but otherwise all prediction intervals around this time are in agreement with the reported values or overshoot them. Similarly, the renewal model greatly undershoots some of the reported case counts (Figure 2A) and then jumps sharply upward the following week.
How much of this week-by-week change in bias might be due to reporting delays and incomplete data?
It would be great to see how the reported case and death numbers evolved over time (e.g., a time-lapse plot of the cases and deaths time-series in each country), to help the reader understand how the reported data may have affected the mechanistic models and the participants.

Thank you for highlighting this. There were indeed a few data updates (the most notable update happened in Germany in October 2020), but most of them did not substantially alter the data. In particular, most updates did not have an effect on weekly numbers (which were the ones relevant for scoring as well as for the crowd forecasts). To make this clearer to the reader we have added a plot comparing data as of then with data as of now and added the following statement in Section 4.1: “*The ECDC data as well as the data published by the Polish Ministry of Health were also subject to data revisions, although most of them (with a notable exception of a data update for October 12 2020 in Germany) only affected daily, not weekly data (see Figures S7 and S8.”* Indeed data revisions are only one particular form of data anomalies and that all models (in particular the ones operating on daily data) are affected e.g. by unobserved factors such as reporting delays.

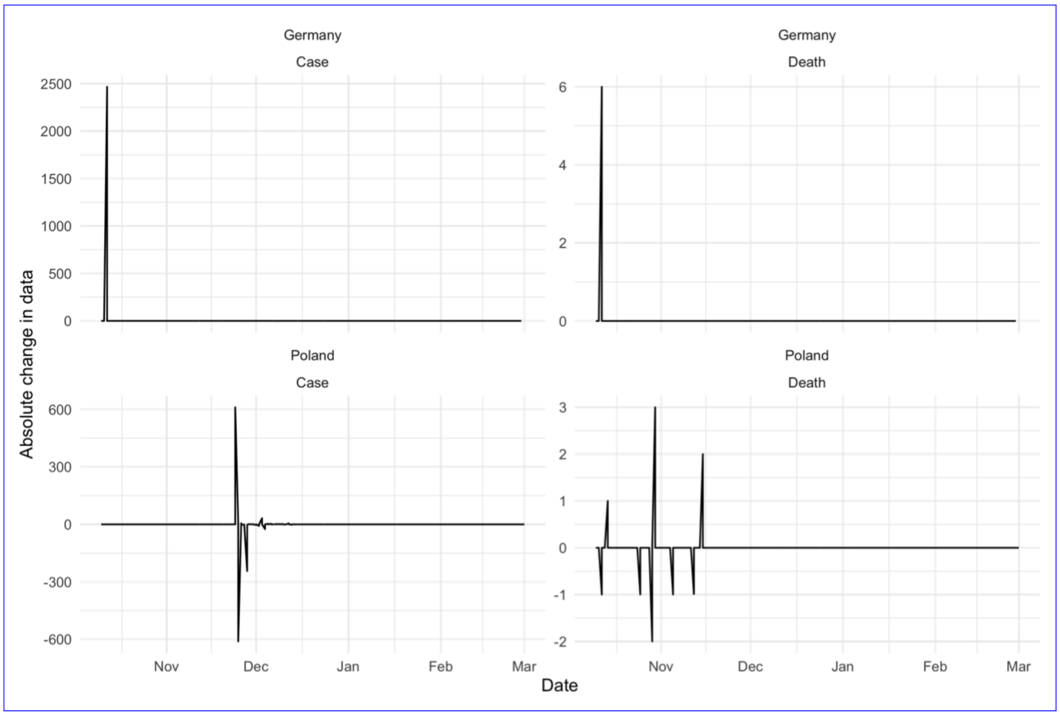

Figure S7: Visualisation of the absolute difference between the daily report data at the time and the data now. In Germany, there were zero cases and deaths reported on 2020-10-12, and only later 2467 cases and 6 deaths were added. Data were last accessed through the German and Polish Forecast Hub on May 10 2022.

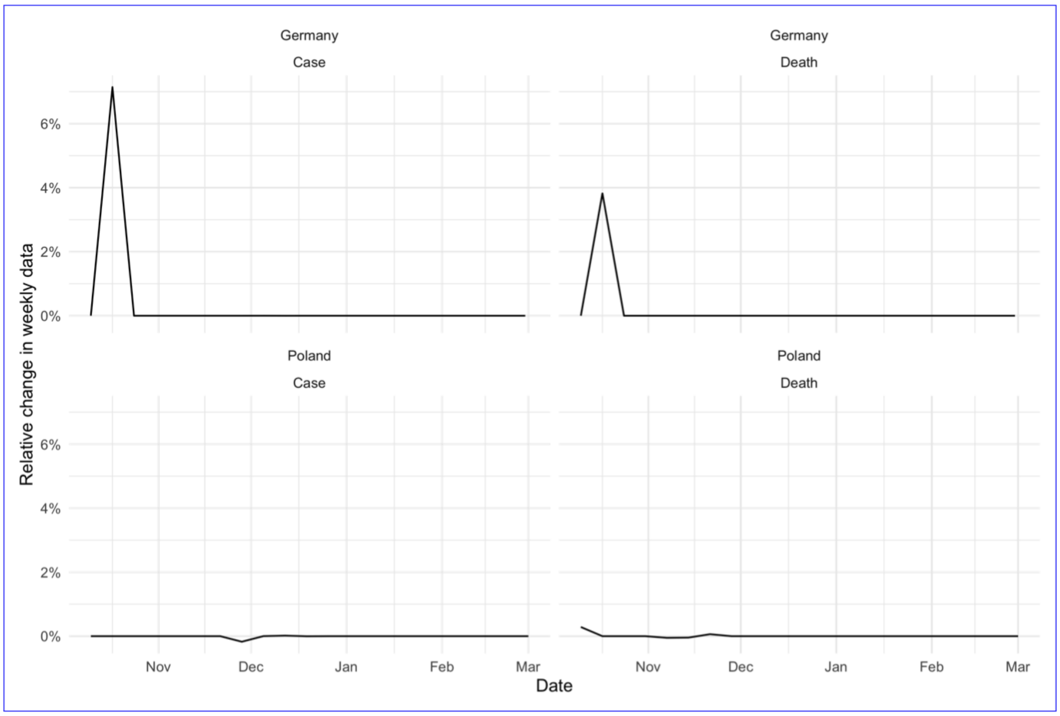

Figure S8: Visualisation of the relative difference between the weekly report data at the time and the data now. Apart from the data that was retrospectively added on 2020-10-12, data updates did not have a noticeable effect on weekly data (as shown in the forecasting application). Data were last accessed through the German and Polish Forecast Hub on May 10 2022.

3. Is it fair to suggest that the performance of the crowd forecasts for case counts, relative to the renewal model, was perhaps driven as much by their overconfidence as it was by their smaller bias?
The crowd forecasts had smaller dispersion (Figure 1E) and fewer WIS outliers (Figures 3 and S8-S10), and the median forecasts had smaller absolute errors than the model-based forecasts (Figure 1G).

Yes, that is a good characterisation. We added the following sentence at the end of the 3rd paragraph in Section 5.2 to make that clearer to the reader: “*The crowd forecasts, on the other hand, showed a smaller bias than the renewal model, but were overconfident.*”

4. It would be great to see alternate versions of Figure 1 provided in the supplementary materials that present performance metrics separately for Germany and Poland.
This would help support some of the observations in the result sections, such as "relative to the Hub ensemble, the crowd forecasts performed noticeably better in Germany than in Poland and the renewal model better in Poland than in Germany" and "In general, there were fewer large outlier forecasts in Poland and in particular the renewal model performed more in line with other forecasts there."

We added two plots to the SI as well as a reference to them at the end of Section 5.2.

Indeed, the renewal model appears to have performed substantially worse in Germany than in Poland (Figure 2A, 2B, and S5-S7).
After the November peak in Poland it appears that it may even have out-performed the crowd forecasts?

Largely owing to the large outlier in January, the renewal model still performed worse than the crowd forecasts even if only taking into account data from December onwards.

The following Figure summarises scores for the renewal model and the crowd forecasts (using only forecasts made after December 1st 2020)

GM case

	interval_score	dispersion	underprediction	overprediction	coverage_deviation	bias	ae_median
Renewal	36700	5960	12400	18300	-0.144	0.138	45500
Crowd forecast	15100	3420	4000	7670	-0.104	0.166	20800

GM death

	interval_score	dispersion	underprediction	overprediction	coverage_deviation	bias	ae_median
Renewal	759	189	142	428	-0.0954	0.162	1090
Crowd forecast	538	146	129	263	-0.124	0.25	853

PL case

	interval_score	dispersion	underprediction	overprediction	coverage_deviation	bias	ae_median
Renewal	11800	3410	5350	3000	-0.104	-0.108	16600
Crowd forecast	8010	2470	3180	2360	-0.118	-0.125	12100

PL death

	interval_score	dispersion	underprediction	overprediction	coverage_deviation	bias	ae_median
Renewal	290	121	124	44.3	-0.0157	-0.198	411
Crowd forecast	199	68.3	74.7	56.3	-0.0446	0.0208	298

Code to reproduce (note: this uses scoringutils version 1.0.0, in contrast to the rest of the paper which uses scoringutils version 0.8.0):

```
install_github("epiforecasts/covid.german.forecasts")
library(dplyr)
covid.german.forecasts::filtered_data |>
  filter(forecast_date > "2020-12-01",
         model %in% c("Renewal", "Crowd forecast"),
         horizon == 2) |>
  score() |>
  summarise_scores(by = c("model", "target_type", "location")) |>
  summarise_scores(fun = signif, digits = 3) |>
  plot_score_table(by = c("location", "target_type")) +
  facet_wrap(location ~ target_type)
```

5. Indeed, an earlier remark about the German forecasts suggests that human insight may have played an important role in the crowd forecasts:

"[H]uman forecasters, possibly aware of the semi-lockdown announced on November 2nd 2020 (50) and the change in the testing regime (with stricter test criteria) on November 11th 2020 (36), were fastest to adapt to the new trend, and the Hub ensemble slowest."

Since 2 of the participants were authors of this study, are the authors able to comment whether they (and potentially other participants) were aware of these announcements when they submitted their predictions?

And how many participants submitted predictions in the relevant weeks of November?

Figure S22 in the SI shows the number of forecasters for every week. In October and November, there were 4-6 forecasters. As far as we can reconstruct it now, all authors were aware that rules would likely be tightened in the near future at least in Germany. However, at least the authors did not take the change in the testing regimes explicitly into account. Generally, the authors were likely more aware of the situation in Germany than in Poland.

1. Figure 1: consider including zero in the y-axis scales for 1E, 1G, and 1H.

Good suggestion, thank you. We updated the Figures accordingly.

2. §5.3 (Death Forecasts, page 15): "The convolution model had a strong tendency to under-predict, which steadily decreased for longer forecast horizons."
The magnitude of the under-prediction decreased (i.e., the bias decreased but the value itself increased).
This could perhaps be worded more clearly, to avoid confusion about under-prediction growing larger for longer horizons.

You are right, thank you. The sentence now reads: "The convolution model had a strong tendency to under-predict for shorter forecast horizons, with the magnitude of under-prediction steadily decreasing for longer forecast horizons."

3. Figure 3: what do the black squares and circles?
I suspect that they represent mean (circle) and median (square) values, but the figure legend and caption do not mention them.

Yes. We updated the Figure caption to make this clear.

4. Figure 4: consider adding an x-axis label (e.g., "Forecast horizon (weeks)"), and using consistent y-axis scales for sub-figures (e.g., the same scale for 4A and 4B, and same scales for case and deaths in each of 4C-4H).

Excellent points, we have updated the plots accordingly.

5. Figure 4: I think that the caption contains several mistakes.
The panels look to be consistent with those in Figure 1, but the Figure 4 caption provides different descriptions for most panels.
For example, panels A and B appear to show mean WIS values, and panels C and D show empirical coverage of different intervals.

Thank you. We have corrected the Figure caption for Figure 4.

6. The number of models in the "Hub ensemble" (i.e., all models except those presented in this manuscript) is explained in §5.4 (page 18), but it could instead be mentioned in §4.4. While reading through the result sections §5.1-5.3 I kept wondering how many models w ere in the ensemble.

We agree this was unclear and have added a line and a reference to the relevant section, table and figure in Section 4.4.

7. The impact of including the convolution model in ensemble forecasts (§5.4) is indeed interesting!
I gather that the convolution model dragged down the "Hub-ensemble-with-convolution" forecasts, resulting in a larger negative bias than for the other ensembles (Figure 4F), but reducing the absolute error of the median forecast (Figure 4G).
Presumably the increased bias reflects outlying WIS values (as evident in Figures 3 and S8-S10)?
From Figures 2 and S5-S7, it seems plausible that the outlying WIS values for the convolution model were underestimates, primarily occurring in Germany over the month of January.

Yes, it seems the effect of the convolution model was to

- Reduce dispersion of the ensemble a bit (Figure 4E)
- Introduce a consistent relative *tendency* towards underprediction (Figure 4F)
- Reduced errors from over-prediction in *absolute terms* (Figure 4B)
- Did increase errors from under-prediction in *absolute terms* a bit, but not much (Figure 4B)
- Reduced absolute error of the median forecasts (Figure 4 G)

The bias value is usually slightly more reflective of a general tendency to over- or under-predict, as it is bounded and only takes into account how much of the predictive probability mass was below or above the observed value. The over- and under-prediction components are usually more affected by outlier predictions, as they take into account the absolute amounts of over- and under-prediction.
